# Reward modulates visual responses in the superficial superior colliculus of mice

**DOI:** 10.1523/JNEUROSCI.0089-23.2023

**Published:** 2023-10-19

**Authors:** Liad J. Baruchin, Matteo Alleman, Sylvia Schröder

**Affiliations:** 1 School of Life Sciences, University of Sussex, Brighton BN1 9QG, UK; 2 UCL Institute of Ophthalmology, University College London, London WC1E 6BT, UK

## Abstract

The processing of sensory input is constantly adapting to behavioral demands and internal states. The drive to obtain reward, e.g., searching for water when thirsty, is a strong behavioral demand and associating the reward with its source—a certain environment or action—is paramount for survival. Here, we show that water reward increases subsequent visual activity in the superficial layers of the superior colliculus (SC), which receive direct input from the retina and belong to the earliest stages of visual processing. We trained mice of either sex to perform a visual decision task and recorded the activity of neurons in the SC using two-photon calcium imaging and high-density electrophysiological recordings. Responses to visual stimuli in around 20% of visually responsive neurons in the superficial SC were affected by reward delivered in the previous trial. Reward mostly increased visual responses independent from modulations due to pupil size changes. The modulation of visual responses by reward could not be explained by movements like licking. It was specific to responses to the following visual stimulus, independent of slow fluctuations in neural activity and independent of how often the stimulus was previously rewarded. Electrophysiological recordings confirmed these results and revealed that reward affected the early phase of the visual response around 80 ms after stimulus onset. Modulation of visual responses by reward, but not pupil size, significantly improved the performance of a population decoder to detect visual stimuli, indicating the relevance of reward modulation for the visual performance of the animal.

## Introduction

The superior colliculus (SC) is a sensory-motor area in the mammalian midbrain with a remarkable diversity of functions (Basso and May, 2017; Wheatcroft et al., 2022). Its superficial layers (sSC) are mainly visual in nature and receive direct input from the retina – from more than 85% of retinal ganglion cells in the mouse (Ellis et al., 2016) – as well as from visual cortex (Cang et al., 2018). Activity in the sSC has been directly linked to specific innate behaviors controlled through projections to specific brain areas outside the sSC (Oliveira and Yonehara, 2018; Liu et al., 2022), e.g., flight or freezing (Shang et al., 2015), and approach behaviors (Hoy et al., 2019). Intermediate and deep layers of the SC integrate input from the sSC and many other brain areas (Isa, 2002; May, 2006; Gale and Murphy, 2014) to control orienting behaviors like directing gaze as well as spatial attention and spatial decisions (Felsen and Mainen, 2012; Zénon and Krauzlis, 2012; Basso and May, 2017; Wang et al., 2020a; Jun et al., 2021; Wang et al., 2022). Understanding visual processing in the sSC is thus an essential prerequisite to understanding the multitude of behaviors that are controlled by the SC.

Despite its early stage in visual processing, activity in the sSC is modulated by the animal’s locomotion and pupil-linked arousal leading to changes in baseline activity, gain modulation of tuning curves for orientation and direction of movement, and changes in preferred spatial frequencies (Joshi et al., 2016; Ito et al., 2017; Savier et al., 2019; Schröder et al., 2020). Similar modulations by ongoing behavior have been observed in the primary visual cortex (V1) (McGinley et al., 2015; Batista-Brito et al., 2018) and other brain areas in many species (Busse et al., 2017; Kaplan and Zimmer, 2020). Modulation by locomotion and pupil-linked arousal in V1 mostly leads to increases in visual responses and thus to improved stimulus decoding (Dadarlat and Stryker, 2017; Christensen and Pillow, 2022). It has been suggested that the observed modulation improves visually dependent behaviors when the animal is running or in a heightened state of arousal (Abdolrahmani et al., 2021; Matteucci et al., 2022; Sörensen et al., 2022). In contrast, modulation by locomotion and pupil-linked arousal in the sSC leads to increases and decreases in visual responses in almost equal numbers of neurons (Savier et al., 2019; Schröder et al., 2020). The purpose of this modulation is unclear at this point and may be linked to differences in the functionality of the modulated cells. These previously observed behavioral and state-dependent modulations inspired us to ask whether the non-visual modulation of sSC extends to other behaviors.

Here we show that reward immediately preceding the presentation of a visual stimulus modulates visual responses in the sSC. Almost all modulated sSC neurons increased responses after the reward, which led to an improvement of stimulus detectability. This modulation was independent of pupil-linked arousal and movements.

## Materials and Methods

All procedures were conducted in accordance with the UK Animals Scientific Procedures Act (1986) under personal and project licenses released by the Home Office following appropriate ethics review.

### Experimental Design and Statistical Analysis

For two-photon imaging, we used 6 mice. We obtained 4 mice (3 male, 1 female) by crossing Gad2-IRES-Cre (www.jax.org/strain/010802) and Ai9 (www.jax.org/strain/007909). The heterozygous offspring expressed TdTomato in glutamate decarboxylase 2-positive (GAD2+) cells. 2 mice (1 male, 1 female) were of the Gad2-IRES-Cre line (www.jax.org/strain/010802). For electrophysiological recordings, we used 3 inbred C57BL/6J mice (www.jax.org/strain/000664; 2 male, 1 female) and 3 mice (3 male) obtained by crossing PV-Cre (www.jax.org/strain/008069) and Ai32 (RCL-ChR2(H134R)/EYFP, www.jax.org/strain/012569). The heterozygous offspring expressed ChR2 in parvalbumin-positive cells, however, no optogenetic manipulations were performed. Animals were 6-29 weeks old at the time of surgery with weights between 20.0 to 43.2 g. They were used for experiments up to the age of 77 weeks. Mice were kept on a 12-h light: 12-h dark cycle. Most animals were single housed after the first surgery. More details for each recording session are listed in Table 1.

If not otherwise stated, single neurons were used as experimental units and results were reported and plotted (lines and shades, circles and error bars) as mean ± standard error of the mean (SEM). We controlled for dependencies across neurons recorded during the same time or belonging to the same animal by using linear mixed-effects models (LMMs) with session and animal as random variable.

### Surgery

Animals were anesthetized with isoflurane (Merial) at 3.5% for induction, and 1-2% during surgery. Carprofen (5 mg/kg; Rimadyl, Pfizer) was administered subcutaneously for systemic analgesia, and dexamethasone (0.5 mg/kg; Colvasone, Norbrook) was administered as an anti-inflammatory agent to prevent brain swelling. The scalp was shaved and disinfected, and local analgesia (Lidocaine, 6 mg/kg, Hameln pharmaceuticals ltd) was injected subcutaneously under the scalp prior to the incision. Eyes were covered with an antibiotic eye-protective gel (Chloramphenicol, Martindale Pharmaceuticals Ltd). After the animal was placed into a stereotaxic apparatus (5% Lidocaine ointment, TEVA UK, was applied to ear bars), the skin covering and surrounding the area of interest was removed, and the skull was cleaned of connective tissue. A custom made headplate was positioned above the area of interest and attached to the bone with Superbond C&B (Sun Medical). Throughout all surgical procedures, the animal was kept on a heating pad to stabilize body temperature at 37°C. Subcutaneous injections of 0.01 ml/g/h of Sodium Lactate (Hartmann’s solution) were given. After the surgery, the animal was placed into a heated cage for recovery from anesthesia. Mice were given three days to recover while being treated with Carprofen.

In animals used for two-photon imaging, a circular 4 mm craniotomy (centered at approximately 4.2 mm AP and 0.5 mm ML from Bregma) was made using a biopsy punch (Kai medical) and a fine-tipped diamond drill (Type 250-012 F, Heraeus). To reduce bleeding from the bone and from the dura we used bone wax and gel foam, and we cooled the area by applying cold cortex buffer. As the posterior SC is covered by a large sinus running through the dura, we permanently pushed the sinus anteriorly to gain optical access to the SC. We first made a cut into the dura directly posterior to the transverse sinus spanning the whole width of the craniotomy. Then we inserted a custom-made implant into the cut and pushed it anteriorly and a few 100 microns down to apply some pressure on the brain and thus stabilize the imaging. The implant was made of a short tube (2.29 mm inner diameter, 1 mm length) made of stainless steel (MicroGroup, Medway, Massachusetts). A 3 mm glass coverslip (#1 thickness, World Precision Instruments) was glued onto the tube to seal the end that was inserted into the craniotomy. A stainless-steel washer was glued onto the other end of the tube. The washer had an inner diameter that fit exactly around the tube and an outer diameter of 5 mm (Boker’s, Minneapolis, Minnesota). All three pieces were glued to each other using a UV curing adhesive (NOA61, Thorlabs). The glass coverslip was slightly larger than the outer diameter of the tube so that it could be slipped underneath the dura. The implant was placed into the craniotomy so that the washer was sitting on top of the skull and provided stability for the implant. The implant was fixed to the skull with Vetbond (3M) and Superbond C&B (Sun Medical). To prevent any dirt from staining the glass coverslip, we filled the tube of the implant with Kwik-Cast (World Precision Instruments), which could be easily removed before imaging.

For two-photon calcium imaging of activity in SC neurons, we injected the virus AAV2/1.Syn.GCaMP6f.WPRE.SV40 (Addgene #100837-AAV1) at a final concentration of 2.30-4.39e12 GC/ml after making the cut into the dura. For the two Gad2-IRES-Cre mice, we additionally injected AAV2/1.CAG.FLEX.tdTomato.WPRE.bGH (Addgene #28306-AAV1). 115-138 nl of the virus(es) were injected 300-500 μm below the brain surface of the SC. The virus was injected at a rate of 2.3 nl every 6 s (Nanoject II, Drummond). The injection pipette was kept in place for about 10 min after the end of the injection.

For electrophysiological recordings, craniotomies were centered above the SC, -4.16 mm AP and 1.0 mm ML from Bregma, and performed the day before the first recording session (in one case 3 h before). The site of the craniotomy was covered with Kwik-Cast (World Precision Instruments) to protect the site from infections. The 3D printed well, which was attached to the skull and surrounded the craniotomy, was closed with a matching 3D printed lid and Blu Tack.

### Animal training

The training procedure has been described in detail previously (Burgess et al., 2017). After at least four days of recovery after surgery, mice were acclimatized to being handled and head-fixed on the experimental apparatus for at least three days. Before training, they were placed on a water control schedule, in which they received at least 40 ml/kg/day. During training, mice received about 3 μl of water at the end of each correct trial. After the daily training, they received top-up fluids to achieve the minimum daily rate. Body weight and potential signs of dehydration were monitored daily, and mice were given free access to water when they were dehydrated, or their weight fell below threshold. The training started with a simplified version of the task involving only high-contrast stimuli and longer response times. As performance improved, lower contrast stimuli were introduced and response times were reduced.

### Experimental apparatus

The experimental apparatus was described in detail before (Burgess et al., 2017). The mouse was head-fixed and surrounded by three computer screens (for two-photon imaging: Iiyama ProLite E1980SD placed ~20 cm from the mouse’s eyes; for electrophysiology: Adafruit, LP097QX1 placed ~11 cm from the mouse’s eyes; 60 Hz refresh rate for both models) at right angles covering approximately 270 x 70 degrees of visual angle. In some experiments, Fresnel lenses (BHPA220-2-6 or BHPA220-2-5, Wuxi Bohai Optics) were mounted in front of the monitors to compensate for reduction in luminance and contrast at steeper viewing angles relative to the monitors. In some of these experiments, lenses were coated with scattering window film (frostbite, The Window Film Company) to prevent specular reflections. A wheel was placed below the mouse’s forepaws and a rotary encoder (Kübler) measured the rotational movements of the wheel. A plastic tube for water delivery was placed in front of the mouse’s snout and calibrated amounts of water were released using a solenoid valve. Licking behavior was monitored by attaching a piezo film (TE Connectivity, CAT-PFS0004) to the plastic tube and recording its voltage. A detailed parts list of the apparatus can be found at http://www.ucl.ac.uk/cortexlab/tools/wheel. To track pupil size, we illuminated one of both eyes with an infrared LED (850 nm, Mightex SLS-0208-A or Mightex SLS-0208-B). Videos of the eye were captured at 30 Hz with a camera (DMK 23U618 or DMK 21BU04.H, The Imaging Source) equipped with a zoom lens (Thorlabs MVL7000) and a filter (long-pass, Thorlabs FEL0750; or band-pass, combining long-pass 092/52x0.75, The Imaging Source, and short-pass FES0900, Thorlabs).

### Behavioral tasks

The task in the two-photon imaging experiments closely followed the paradigm described earlier (Steinmetz et al., 2019). The task paradigm has previously been termed *two-alternative unforced choice task* and combines elements of two-alternative forced choice tasks (animal needs to detect a stimulus on the left or right side of its visual field) and Go-NoGo tasks (animal has to refrain from an action when no stimulus is presented). To start a trial, the mouse had to keep the wheel still for 0.5-3 s (randomly chosen from uniform distribution). At initiation of 60-90% of all trials, a visual stimulus was presented at 95-115 deg either to the left or the right of the vertical meridian. The stimulus was a Gabor patch with vertical orientation (90 deg), sigma of 9 deg, spatial frequency of 0.1 cycles per deg and contrast of 12.5-100% (the range of presented contrasts depended on each animal’s task performance). In the rest of the trials, no stimulus was presented. After 0.3-1.2 s (randomly chosen from uniform distribution), an auditory go cue (8 kHz for 0.2 s) signaled that the position of the stimulus (if present) was now coupled to the wheel movement. The time count between stimulus onset and go cue was reset if the mouse moved the wheel during this period. After the go cue, the mouse had to either move the presented stimulus towards the vertical meridian (to 50-60 deg from the vertical meridian) or, if no stimulus was presented, to hold the wheel still (more precisely: not cross the threshold for moving a stimulus to the target position). The mouse had to perform its choice within a fixed interval (“response time”) of 1.5-2 s after the go cue. After a correct choice, the mouse was given a water reward of 2-3 μl as soon as the stimulus reached its target position or at the end of the response time. The visual stimulus (if present) disappeared 1 s after reward delivery. After an incorrect choice, i.e., moving the stimulus by 50-60 deg into wrong direction (towards the periphery), not reaching the target position within the response time, or moving the wheel too far while no stimulus was presented, an auditory white noise stimulus was played for 2 s, after which the visual stimulus (if present) disappeared. The mouse could then start the next trial. Trials of different contrast conditions were randomly interleaved. However, the same condition was repeated when the mouse responded incorrectly.

For two of six mice, the task paradigm had the following differences: (1) There were no NoGo trials where no stimulus was presented. (2) In every trial, two stimuli of different contrasts were presented, one in the left and one in the right visual field, and the animal had to move the higher contrast stimulus towards the center. (3) The response time was unlimited. The task paradigm for these animals can be described as a *two-alternative forced choice task*.

The task in the electrophysiology experiments was very similar to the task described at the beginning of the section (two-alternative unforced choice task). The only differences were the following: (1) The mouse had to keep the wheel still for 0.2-0.5 s to start a trial. (2) Two Gabor patches were presented simultaneously, one to the left and one to the right, at 26-80 deg from the vertical meridian. The mouse had to choose the patch with the higher contrast and move it to the vertical meridian. (3) The width of the Gabor patches (sigma) varied between 8-11 deg and contrasts varied between 25-100%. (4) Any wheel movements between stimulus onset and go cue were simply ignored. (5) The go cue appeared 0.4–0.8 s after stimulus onset. (6) The negative feedback (auditory white noise) was played for 1.5-2 s. (7) The response time was 1.3-1.6 s long.

### Two-photon imaging

Two-photon imaging was performed using a standard resonant microscope (B-Scope, ThorLabs Inc.) equipped with a 16x, 0.8 NA water immersion objective (N16XLWD-PF, Nikon) and controlled by ScanImage 4.2 (Pologruto et al., 2003). Excitation light at 970-980 nm was delivered by a femtosecond laser (Chameleon Ultra II, Coherent). Multi-plane imaging was performed using a piezo focusing device (P-725.4CA PIFOC, Physik Instrumente, 400 μm range). Laser power was depth-adjusted and synchronized with piezo position using an electro-optical modulator (M350-80LA, Conoptics Inc.). The imaging objective and the piezo device were light shielded using a custom-made metal cone, a tube, and black cloth to prevent contamination of the fluorescent signal caused by the monitors’ light. Emission light was collected using two separate channels, one for green fluorescence (525/50 nm emission filter) capturing the calcium transients and one for red fluorescence (607/70 nm emission filter) capturing the expression of TdTomato in inhibitory neurons of Gad-Cre x TdTomato mice.

For imaging neurons in SC, we used 2-4 imaging planes separated by 30-55 μm at depths of 12-260 μm from the surface of SC. The field of view spanned 410-770 μm in both directions at a resolution of 512 x 512 pixels. The frame rate per plane was 6-10 Hz. For one dataset, a single plane was captured at a frame rate of 30 Hz.

### Electrophysiology

Recordings were made using Neuropixels 1.0 electrode arrays (Jun et al., 2017). Probes were mounted to a custom 3D-printed piece and affixed to a steel rod held by a micromanipulator (uMP-4, Sensapex Inc.). Prior to insertion, probes were coated with a solution of DiI (ThermoFisher Vybrant V22888 or V22885) or DiO (ThermoFisher Vybrant V22886) by holding 2 μl in a droplet on the end of a micropipette and touching the droplet to the probe shank, letting it dry, and repeating until the droplet was gone. Probes had a soldered connection to short external reference to ground; the ground connection at the headstage was subsequently connected to an Ag/AgCl wire positioned on the skull. The craniotomies and the wire were covered with saline-based agar. The agar was covered with silicone oil to prevent drying. In some experiments a saline bath was used rather than agar. Probes were advanced through the agar and the dura, then lowered to their final position at ∼10 μm/sec. Electrodes were allowed to settle for ∼10 min before starting recording. Recordings were made in external reference mode with LFP gain = 250 and AP gain = 500. Data were filtered in hardware with a 300 Hz 1-pole high pass filter and digitized at 30 kHz. Recordings were repeated at different locations on each of multiple subsequent days.

### Pre-processing of imaging data

All raw two-photon imaging movies were analyzed using suite2p (implemented in MATLAB, Mathworks) to align frames and detect regions of interest (Pachitariu et al., 2016). We used the red channel representing TdTomato expressed in all inhibitory neurons to align frames, which yielded better results than alignment using calcium dependent fluorescence. Every aligned movie was inspected manually to check for failures in automatic alignment. Failures were corrected using different parameter settings where possible. Misaligned movie frames were discarded and ROIs in unstable regions of the field of view were not considered for further analysis.

Using the aligned movies and detected ROIs resulting from suite2p analysis, we extracted the fluorescence from the green and the red channel within each ROI. To correct the calcium traces for contamination by surrounding neuropil, we also extracted the fluorescence of the surrounding neuropil for each ROI using the green channel. The neuropil mask resembled a band surrounding the ROI with its inner edge having a distance of 3 microns away from the edge of ROI and its outer edge having a distance of 30 microns from the edge of the ROI. Pixels belonging to other ROIs were excluded. To correct for contamination, the resulting neuropil trace, N, was subtracted from the calcium trace, F, using a correction factor α: F_c_(t) = F(t) – α·N(t). The correction factor was determined for each ROI as follows. First, F and N were low-pass filtered using the 8^th^ percentile in a moving window of 180 s, resulting in F_0_ and N_0_. The resulting traces F_f_(t) = F(t)-F_0_(t) and N_f_(t) = N(t)-N_0_(t) were then used to estimate α as described previously (Dipoppa et al., 2018). In short, N_f_ was linearly fitted to F_f_ using only time points when values of F_f_ were relatively low and thus unlikely to reflect neural spiking. F_c_ was then low-pass filtered as above (8^th^ percentile in a moving window of 180 s) to determine F_c,0_. These traces corrected for neuropil contamination were then used to determine ΔF/F = (F_c_(t) – F_c,0_(t)) / max(1, mean(F_c,0_(t)).

To correct for potential brain movements, we used the red fluorescence traces of each ROI to regress out changes in fluorescence that were not due to neural activity. First, we low-pass filtered the red trace of each ROI (8^th^ percentile in a moving window of 180 s) and subtracted it from the unfiltered trace to remove slow drifts and bleaching effects. Second, we applied a median filter to the resulting red trace (moving median in window of 10 s). Third, this trace was regressed out of ΔF/F.

We avoided sampling the same neurons more than once. We detected ROI pairs that were close to each other in neighboring imaging planes and that had highly correlated calcium traces (ρ > 0.4, correlation between traces filtered using a moving median in a window of 5 samples). Only the ROI of each pair with the highest signal-to-noise ratio was used for further analyses. ROIs that had very long-lasting calcium transients (> 25 s) were removed.

### Spike sorting

Extracellular voltage traces were pre-processed using common-average referencing: subtracting each channel’s median to remove baseline offsets, then subtracting the median across all channels at each time point to remove artifacts. Electrophysiological data collected in SC was spike sorted using Kilosort2.5 (available at https://github.com/MouseLand/Kilosort/releases/tag/v2.5) with standard parameters (Pachitariu et al., 2023). After sorting, all automatically detected spike clusters were curated manually using phy (https://github.com/kwikteam/phy). The curation followed strict and consistent guidelines. Clusters with the following characteristics were discarded: 1) <1,000 spikes for the entire recording duration and small spike amplitudes (<3x recording noise) or any spikes within ±2 ms of the spike auto-correlogram, 2) atypical waveform or waveform looks like the sum of two or more waveforms, 3) spike amplitudes <2x recording noise, 4) spike amplitude changed over time and reached the detection threshold, 5) firing rate abruptly changed, which could not be explained by a change in experimental paradigm, 6) spike rate within ±2 ms of the spike auto-correlogram is higher than half the mean firing rate (tails of auto-correlogram) or reaches the level of the mean firing rate more than once. We then quantified the quality of our selected single units (mean ± SEM). First, we measured the average spike amplitude of the average spike shape: 136.02 μV ± 2.2 μV. Then, we quantified for each unit how many of its spikes fall in the 2 ms inter-spike interval as a percentage of its total spikes: 0.8% ± 0.09%. To quantify possible drift, we divided each session into 100 equal segments and counted in how many segments the unit spiked: 81% ± 0.5%.

### Location of electrophysiological recordings

We used the LFP recorded on each channel of the Neuropixels probes to determine the surface of the SC (Ito et al., 2017). We used LFP responses to the following visual noise stimulus: We presented white squares of 8 visual degrees edge length, positioned on a 10 × 36 grid (some stimulus positions were located partially off-screen) on a black background. The presentation of each white square lasted on average 6 monitor frames (100 ms), and their times of appearance were independently and randomly selected to yield an average rate of approximately 0.12 Hz. We then determined the stimulus square that elicited the most negative LFP amplitudes based on the average LFP waveform triggered by a change of luminance in each square. At the time of peak negative LFP amplitude measured on the probe channel with the most negative peak, we calculated the LFP amplitude as a function of depth across all probe channels separately. We then determined the depth at half height of the negative peak (located above the depth of the peak) for all right and left channels on the probe. The average result of the right and left probe channels was defined as the surface of the SC.

To determine the depth of SC along the probe and the border between sSC and dSC, we reconstructed the probe location on the basis of histological brain slices. Mice were perfused with 4% PFA, the brain was extracted and fixed for 24 hours at 4 C in PFA, then transferred to 30% sucrose in PBS at 4°C. The brain was mounted on a microtome in dry ice and sectioned at 60 μm slice thickness. Sections were washed in PBS, mounted on glass adhesion slides, and stained with DAPI (Vector Laboratories, H-1500). Images were taken at 4x magnification for each section using a Zeiss AxioScan, in three colors: blue for DAPI, green for DiO, and red for DiI. We next used SHARP-Track (Shamash et al., 2018) or AP_histology (https://github.com/petersaj/AP_histology) to align each brain slice to a plane through the Allen Mouse Common Coordinate Framework (http://atlas.brain-map.org/) and record the 3D coordinates of manually selected points along the fluorescence track. A line was fitted through the coordinates, resulting in a vector of brain regions the electrode passed through. The overlay of brain areas onto the brain slices and the vector of brain regions were used to determine the depth of SC and the location of the sSC-dSC border relative to the SC surface.

### Behavioral tracking

#### Pupil size and position

We used DeepLabCut (Mathis et al., 2018) to detect 8 landmarks in our eye videos: top/bottom/left/right edge of the pupil and top/bottom/left/right edge of the eye lids. The trained network is available from https://github.com/sylviaschroeder/PupilDetection_DLC. Pupil diameter was defined as the distance between the top and bottom edges (height) of the pupil. This measure was chosen as it is relatively independent from horizontal eye movements (the most common eye movements mice perform), while the distance between left and right pupil edges (width) changes with eye movements. For time points when the lids covered top or bottom pupil edges (i.e., certainty of top/bottom markers returned by DLC was low or the markers were very close to markers for top/bottom edges of the lid), we used eye position and left and right pupil edges to estimate pupil diameter. Based on samples where the pupil was not covered by the lid, we used local linear regression (Matlab function fit, with method lowess and span = 0.1) to fit pupil height given pupil width and horizontal position of pupil center. Times when the eye was closed, including 5 frames before and after detected closure, were ignored for further analysis. The eye was defined closed when one of the following criteria was true: (1) certainties of left or right pupil marker was low, (2) small distance between top and bottom lid, (3) certainties of top and bottom pupil marker was low, or (4) lid too close to center of pupil. Values of pupil diameter were median filtered with a span of 5 frames. Pupil size per trial was determined by averaging pupil size in a -0.5–0.5 s window from stimulus onset. A trial was considered a large-pupil trial if the average value was above the median pupil size for the session.

#### Whisker movement

During 17 electrophysiology recordings, we also recorded the face of the animal on video. To quantify whisker movement, we used Facemap (Syeda et al., 2022). We placed a region of interest (ROI) on the whisker pad in the video. Facemap then extracts the motion energy in the ROI by performing a singular value decomposition (SVD) on the differences between consecutive frames. We used the first singular vector to estimate the strength of whisker movement.

#### Licking

Lick rate was measured based on the voltage trace recorded from the piezo element that was attached to the reward tube. The trace was z-scored and a lick was detected at every peak larger than 1 standard deviation. In a lick burst only the first lick event was selected and any lick with an interval of less than 200ms was discarded.

### Mapping receptive fields

We presented sparse sequences of white and black squares to characterize the receptive fields, using the responses to white squares for the ON subfield and to black squares for the OFF subfield. We used a linear regression model to simultaneously fit two spatio-temporal filters—one for the ON field and one for the OFF field—covering 0.2-0.4 s before the measured neural response. For the ON field, only the appearance of white squares was considered, for the OFF field only black squares. The fits were regularized by imposing spatial smoothness on the two receptive fields (Smyth et al., 2003). We set the regularization parameter to *λ = 10^-4^* × #parameters where #parameters is the grid size (possible positions of squares) times the duration of the spatio-temporal RF in frames. Using this parameter, the receptive fields were then modelled using the complete calcium trace of each unit in response to the visual noise stimulus. To evaluate model fit, we fitted a two-dimensional Gaussian to the ON-, OFF- and the average of ON- and OFF-subfield at the time of peak response. We continued with the Gaussian fit, *f*, resulting in the minimum mean square error compared to the subfield it was fitted to (ON, OFF, or ON+OFF). Then we determined the optimal temporal kernel, *t*, so that multiplied with the Gaussian fit it best fits the spatio-temporal RF. Finally, we predicted the neural response by convolving the visual noise stimulus with *f* × *t* and determined the explained variance of this prediction. A neuron was considered to have a genuine receptive field if (1) the explained variance was >0.015, and (2) the peak of the Gaussian fit, *f*, was 3.5 times larger than the noise of the RF, i.e., the standard deviation of the difference between the RF (at time of maximum) and the Gaussian fit, *f*.

To quantify the overlap between the RF and the task stimulus, the following formula was used to describe the area (*A*) of intersection:

(1) $$\text{A}=r_{s}^{2}\cos^{-1}\left(\frac{d^{2}+r_{s}^{2}-r_{RF}^{2}}{2dr_{s}}\right)+r_{RF}^{2}\cos^{-1}\left(\frac{d^{2}+r_{RF}^{2}-r_{s}^{2}}{2dr_{RF}}\right)$$

$$-\frac{1}{2}\sqrt{\left(-d+r_{s}+r_{RF}\right)\left(d+r_{s}-r_{RF}\right)\left(d-r_{s}+r_{RF}\right)\left(d+r_{s}+r_{RF}\right)}$$

where *r_s_* and *r_RF_* are the radii of the Gabor stimulus and two-dimensional Gaussian fitted to the RF (radius is mean across both dimensions), calculated as 1 sigma and 1.5 sigma respectively, and *d* is the distance between the centers of the stimulus and RF. This area, *A*, was then divided by the total area of the RF to measure stimulus-RF overlap. Any pair of stimulus and RF that had a distance larger than the sum of radii was considered to have zero overlap.

### Visual responses and modulation

#### Contrast response fitting

Responses to visual stimuli were determined by, first, averaging calcium traces (previously scaled to maximum of 1 and minimum of 0 across the whole recording) across a window from 0-500 ms after stimulus onset for two-photon imaging recordings, and second, subtracting baseline activity, i.e., the average calcium trace 0-500 ms before stimulus onset. For electrophysiology recordings, we summed the number of spikes within the window of 0-200 ms after stimulus onset and divided by the time (200ms). These firing rates were then divided by the maximum firing rate measured by convolving each neuron’s spike times with a Gaussian window of 30 ms across the whole recording. The post-stimulus time windows of 500 ms and 200 ms were chosen based on the peak latency of the visual response recorded by the two methods. These responses (one per trial) were used to fit a hyperbolic ratio function (Albrecht and Hamilton, 1982):

(2) $$\text{f}(c)=\text{R}\frac{c^{n}}{c^{n}+c_{50}^{n}}$$

where *R* is gain, *c* is stimulus contrast, *c*_50_ is contrast at half-saturation, and *n* is rate of change. For the electrophysiological data where two Gabor patches were presented simultaneously only the contrast at the contralateral side was considered while the ipsilateral contrast was ignored because ipsilateral stimuli did not usually influence visual responses. The curve was only fit to neurons that exhibited a significant difference in response amplitude after stimulus onset compared to baseline activity before stimulus onset (see *Responsiveness to task events*). If the fit resulted in an explained variance larger than that of a flat line fitted to the responses, the neuron was considered *visually responsive*. 90 neurons in the two-photon recordings and 92 neurons in the electrophysiology recordings had significant responses to contralateral visual stimuli but could not be fitted well with the hyperbolic ratio function. Only visually responsive neurons were tested for modulation by task variables.

To test for the effect of previous feedback (reward or negative feedback), pupil size (small or large), upcoming action (Go or NoGo), and upcoming outcome (correct or incorrect) on response amplitude, we fitted the individual neuronal responses to function (2) as above, where gain R were defined as follows:

(3) $$R=R_{0}+w_{p}\delta_{p}+w_{f}\delta_{f}+w_{a}\delta_{a}+w_{o}\delta_{o}$$

The factors represented by *w* are weights and *δ* ∈ {0,1} represents the presence or absence of a specific task variable: pupil size (*p*), previous feedback (*f*), action (*a*), or outcome (*o*). *R_0_* is the gain when all *δ* are zero.

To find the best model explaining the visual responses, we fitted variations of this function, leaving out any possible combination of *δs* so that either all or only some of the behavioural conditions would be considered for the modulation of visual responses. The cross-validated explained variance was calculated using a 10-fold cross-validation: the dataset was divided into 10 parts; for each part, the model was first trained on the other 9 parts, then explained variance was determined on the remaining part; the resulting explained variances were averaged across all 10 test parts. We then employed a permutation test to test the significance of each task variable (see *Permutation Test*). The model with the highest cross-validated explained variance that contained only significant task variables (p < 0.05) constituted the best model.

#### Response modulation

Response modulation of visually responsive neurons was calculated using the following formula:

(4) $$RM=\frac{A-B}{0.5(A+B)}$$

where *A* was the gain in either previously rewarded, large pupil, Go, or correct trials; and *B* was the gain in either previously negative feedback, small pupil, NoGo, or incorrect trials.

### Decoding stimulus presence

We used responses, averaged across a 0.5 s time window after stimulus onset, of all neurons in the recorded population to decode the presence or absence of a contralateral visual stimulus. We trained a logistic regression model on 50% of the data, and used the other 50% to test the model (Christensen and Pillow, 2022). The fitted function was:

(5) $$\ln\left(\frac{p}{1-p}\right)=Xb$$

where *p* is the probability that stimulus is present, *X* is the neuronal response matrix [trial × number of neurons], and *b* is the coefficient vector [number of neurons × 1].

We split the test data into two groups. The split was either according to previously positive/negative feedback, large/small pupil, Go/NoGo, or correct/incorrect. We stratified the data so that both splits were of equal size. We repeated the training procedure 10 times and used the mean prediction score of each group for comparison. We scored the prediction using the Matthew Correlation Coefficient (Matthews, 1975). The score ranges from -1 to 1, where 0 is chance level, 1 is a perfect prediction, and -1 is a perfectly opposite prediction. This measure combines prediction accuracy and recall. To control for possible covariance between the task variables (pupil size, previous feedback, action, and outcome), we repeated the procedure described above while keeping one variable constant (e.g., by only considering trials where pupil size is large).

### Decoding trial outcome

To predict trial outcome from the visual stimulus and previous choices, we used a logistic regression model with 7 independent variables: an intercept, a stimulus of 0% contrast on both sides (*Zero* ∈ {0, 1}), a stimulus with left contrast ≥ 0% (*Left* ∈ {0, 0.125, 0.25, 0.5, 0.75, 1}), a stimulus with right contrast ≥ 0% (*Right* ∈ {0, 0.125, 0.25, 0.5, 0.75, 1}), and correct choice on each of the previous 3 trials (*R_T-1_*, *R_T-2_*, *R_T-3_* ∈ {0, 1}). The model was trained on 80% of the data and tested on the remaining 20%. The formula was:

(6) $$\ln\left(\frac{p}{1-p}\right)=a_{0}+a_{1}\mathrm{Zero}+a_{2}\mathrm{Left}+a_{3}\mathrm{Right}+a_{4}R_{T-1}+a_{5}R_{T-2}+a_{6}R_{T-3}$$

where *a*_0_-*a*_6_ are the coefficients and the variables are as described above.

Like for the logistic regression to predict stimulus presence (see previous paragraph), we used the Matthews Correlation Coefficient (Matthews, 1975) to score the model prediction. To measure the significance of the parameter coefficients, we performed a Wald test (Wald, 1943) with the null hypothesis of the parameter being equal to 0.

### Response fluctuation analysis

Response fluctuations of neural response that are independent of visual input can occur across long timescales of many seconds to minutes. To measure response fluctuations across a recording session, we accounted differences in visual drive by different visual contrasts in the following way. For each neuron, response amplitudes to each visual stimulus were calculated. The time windows were the same as those described in *Contrast response fitting*. For each contrast, single trial responses were z-scored by the contrast specific mean and standard deviation. To measure the time scale of fluctuations, we calculated the auto-correlogram of this fluctuation signal. To test if any point in the auto-correlogram is significantly larger than expected, we generated a null distribution by randomly drawing 500 values from the auto-correlogram. We used the 2.5^th^ to 97.5^th^ percentile interval of the null distribution to determine significant values as consecutive lags (from 0) that were outside the interval.

To model the impact of response fluctuations, i.e., trial-to-trial variability, the calculation of contrast gain in equation (2) was modified to include a response fluctuation variable as follows:

(7) $$R=R_{0}+w_{p}\delta_{p}+w_{f}\delta_{f}+w_{a}\delta_{a}+w_{o}\delta_{o}+w_{d}d$$

where *d* is the response fluctuation of the previous trial and *w_d_* is the fitted weight.

### Reward history analysis

We quantified reward history for a stimulus based on a cumulative count of correct minus incorrect choices made in response to the stimulus. We considered all presentations of a contralateral stimulus regardless of contrast. For the n^th^ presentation of the stimulus, reward history increased by 1 if the animal responded correctly and decreased by 1 otherwise, down to a minimum of 0. We avoided negative reward histories as animals did not seem to be actively repulsed from a choice followed by negative feedback. Reward history stayed constant if any other than the contralateral stimulus was presented.

To model the influence of reward history on visual responses, the calculation of contrast gain in equation (2) was modified to include a reward history variable as follows:

(8) $$R=R_{0}+w_{p}\delta_{p}+w_{f}\delta_{f}+w_{a}\delta_{a}+w_{o}\delta_{o}+w_{h}h$$

where *h* is reward history of the current trial and *w_h_* is the fitted weight.

### Responsiveness to task events

We tested responses to the following events: (1) contralateral visual stimulus in trials without wheel movements within test window (to dissociate visual from movement responses), (2) ipsilateral visual stimulus in trials without wheel movements, (3) auditory go cue in trials without wheel movements (to dissociate auditory from movement responses), (4) ipsilateral and contralateral wheel movements in no-stimulus trials (to dissociate wheel from visual movement responses), (5) positive and negative feedback in no-stimulus trials (to dissociate feedback from visual responses), and (6) licks at least 0.5 s outside the reward delivery time to dissociate licking from reward responses. In the case of lick bursts (licks within less than 0.3 s from each other), only the first lick was taken into consideration. We measured average calcium responses or firing rates in windows of 0–500 ms after event onset (post-event responses) and compared these to average responses in windows of 0–500 ms before event onset (pre-event responses) using paired t-tests. For responses to visual stimuli (contra- or ipsilateral, respectively), we used a two-way ANOVA with pre- and post-event time as one factor and stimulus contrast as second factor. A neuron was considered responsive to a task event if p < 0.05, after Bonferroni multiple test correction.

### Permutation Test

Permutation tests were performed by generating surrogate datasets from the recorded data, computing the test statistic for each surrogate dataset, and comparing the resulting null distribution to the test statistic of the recorded data. Surrogate datasets were generated by randomly permuting the values of the task variables, e.g., small and large pupil size. A result was deemed significant at p < 0.05, i.e., if the test statistic measured on the original data was outside the 2.5^th^ to 97.5^th^ percentile interval of the surrogate datasets.

To determine significance of gain changes in the contrast response function due to task variables (Figure 2G-J, Figure 4C, Figure 5J, Figure 6G-H), we generated 200 surrogate datasets per recorded dataset by permuting the values of the task variable (e.g., small and large pupil). For each neuron, we fitted the hyperbolic ratio function to each of the permuted datasets to generate a null distribution of 200 instances of cross-validated (10-fold) variance explained. We used those same functions fitted to the surrogate datasets to derive the null distribution of response modulations and to determine the 2.5^th^ to 97.5^th^ percentile interval of null distribution for each possible value of response modulation (Figure 2K-N, Figure 6I-J).

To determine significance for the performance of the logistic regression model that predicts stimulus presence (Figure 3A-E), we generated 200 surrogate datasets per recorded dataset by permuting the values of the task variable (e.g., previous positive and negative feedback). The test statistic was the difference between prediction scores for the two splits of the test data (according to value of the task variable). In order to determine significance across datasets, the mean across sessions of the actual test statistic was then compared to the distribution of test statistics across the surrogate datasets.

To determine significance for the logistic regression model that predicts behavioral outcome (Figure 3F), we generated 500 surrogate datasets per recorded dataset by permuting the value of the trial outcome, i.e., correct and incorrect choices. Only scores larger than the 95^th^ percentile of the surrogate scores were considered significant.

To determine whether eye positions at time of stimulus onset were significantly different after positive versus negative feedback (Figure 4D), we used the distance of the centers of mass of eye positions for positive versus negative feedback as test statistic. We then generated 200 surrogate datasets by permuting the labels of positive and negative feedback across trials.

## Results

**Figure 1:**
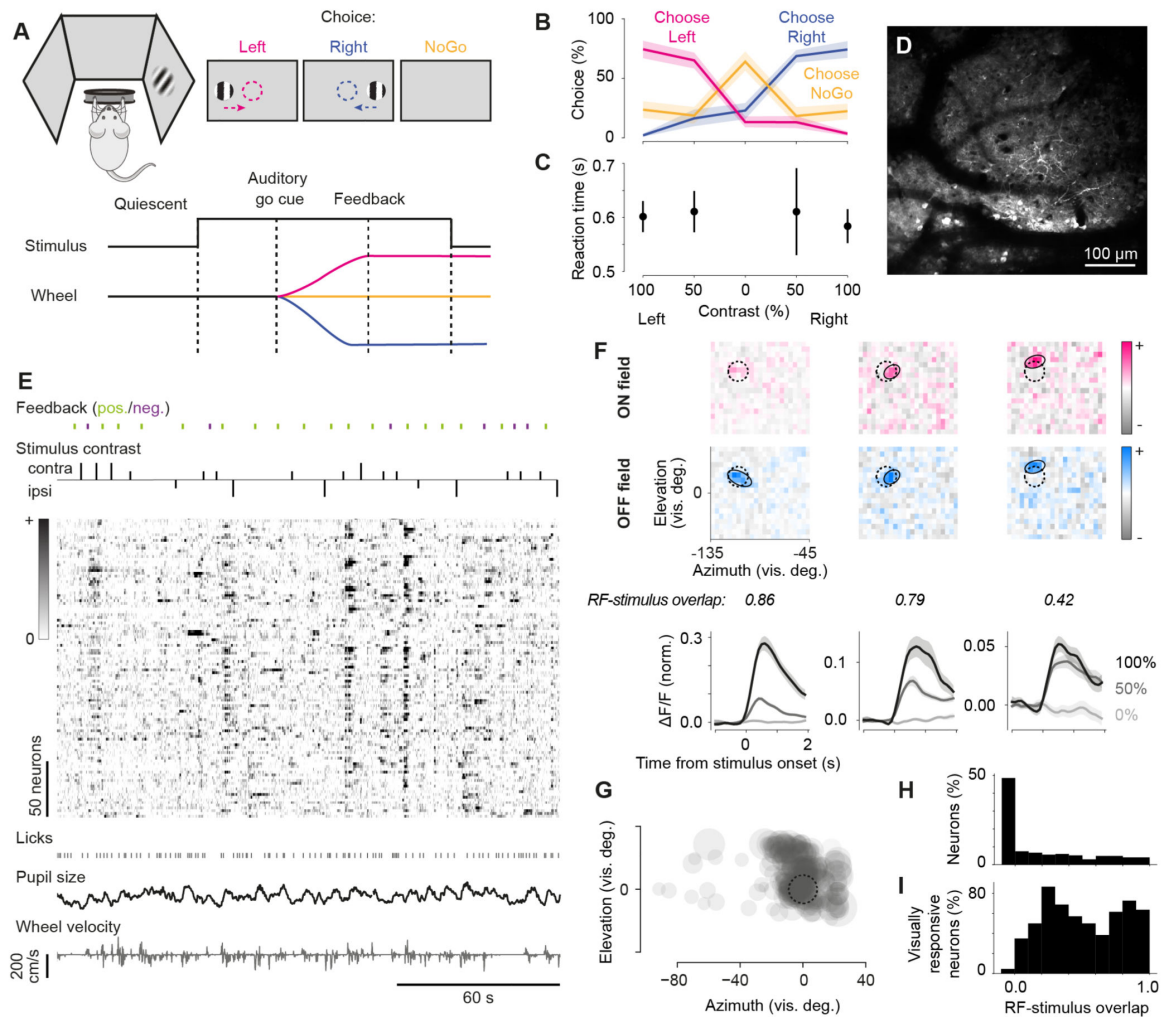
Superficial superior colliculus neurons were imaged during a visual decision task. **A**. Experimental setup (*top left)*, correct choices for various stimulus conditions (*top right*), and time course of each trial (*bottom*). **B**. Psychometric curve. Percentage of left (pink), right (blue), or NoGo (yellow) choices (22 sessions in six mice) depending on stimulus contrast (or difference in contrast between stimuli). Trials in which the animal was disengaged, i.e., ≥3 consecutive NoGo trials, were discarded. Average performance, i.e., percentage of correct choices: 64.9% ± 1.4%. **C**. Reaction time (mean ± SEM across sessions, median per session) measured as time from go cue to time when stimulus reached its target position. Only Go trials were considered. **D**. Average frame showing one imaging plane of one two-photon imaging session (same session as data in **E** and **F**). **E**. Example dataset showing task events (stimulus contrast of 50% or 100%, feedback), calcium traces (z-scored) of 270 simultaneously recorded neurons (sorted by responsiveness to task events, as in Figure 7A), and animal behavior (licks, pupil size, wheel turns). **F**. ON and OFF receptive fields (*top*, continuous outlines show Gaussian fits at 1.5 STDs) of three SC neurons and task stimulus position (dashed outlines at 1 STD of Gaussian stimulus mask). Calcium traces (baseline subtracted) of same neurons to three contrasts of contralateral task stimulus. **G**. Receptive field (RF) positions and sizes (1.5 STDs, mean of short and long STD of elliptic Gaussian fit) of all recorded neurons (for which RF could be mapped) relative to stimulus position (331 neurons, 12 sessions in five mice). **H,I**. RF-stimulus overlap of all neurons with mapped RF (**H**) and all neurons with significant response to task stimulus (**I**).

We trained six mice to perform a visual decision task (Figure 1A-C) where a visual stimulus (Gabor patch) of varying contrast appeared in the left or right hemifield and had to be interactively moved towards the center of the visual field by turning a wheel under the animal’s forepaws. Each stimulus was a circular, static grating of vertical orientation and was convolved with a Gaussian mask (standard deviation of 9°) so it smoothly faded into the grey background. Left and right stimuli were positioned symmetrically at the same elevation (10-20° above height of the eyes) and the same distance from the vertical midline (95-115° azimuth). For four of six mice, the task also included trials where no stimulus was presented, and the wheel had to be held still (*NoGo* action). For the other two mice, the task instead included trials with a stimulus in both hemifields and the higher contrast stimulus had to be moved to the center. Each trial started with a quiescent period where the wheel had to be still for 0.5-2.5 s before the stimulus appeared (Figure 1A). 0.3-1.2 s after stimulus onset, an auditory go cue signaled the mouse to move the stimulus, or to hold the wheel still. If the mouse performed the correct action, it was rewarded with a drop of water. An incorrect action was followed by auditory white noise. Mice were able to learn this task within 2-4 weeks, performing at approximately 75% correct in response to the highest contrast stimuli (Figure 1B) and mean reaction times of approximately 0.5-0.7 s after the auditory go cue (Figure 1C).

Using two-photon imaging, we recorded 3,919 neurons across 22 task sessions from all six mice and found that 497 (13%) of these neurons reliably responded to the contralateral visual task stimulus (Figure 1D-I). In addition to recording neural responses (Figure 1D) and task events, we recorded several behaviors of the animal, i.e., time of licks, pupil size, and wheel turns (Figure 1E). Responsiveness to the visual stimuli (p < 0.05, ANOVA) in the task was in good agreement with the degree of overlap of a neuron’s receptive field (RF) with the contralateral stimulus. For a subset of the recording sessions, we mapped RFs of the neurons using a visual noise stimulus, which consisted of rapid presentations of small white and black squares on a grey background (12 sessions in five mice). Our examples show one neuron with an OFF and two neurons with ON and OFF RFs that largely overlap with the stimulus (Figure 1F). The task stimuli drove all three neurons in a contrast dependent manner. About 50% of the 331 neurons, for which we could map a RF, showed a zero RF-stimulus overlap (Figure 1G,H) and did not respond to the task stimuli (Figure 1I). 58% of neurons with a positive RF-stimulus overlap responded to the task stimulus, while the rest may prefer different stimulus sizes or orientations. In addition, 281 of 2,585 neurons that did not yield a RF in response to the visual noise were responsive to the task stimuli. We will focus further analyses regarding the behavioral modulation of visual responses on the subset of neurons that were visually responsive to the contralateral task stimulus. Unless otherwise stated, the following results will be based on the data recorded in 22 task sessions using six mice.

**Figure 2:**
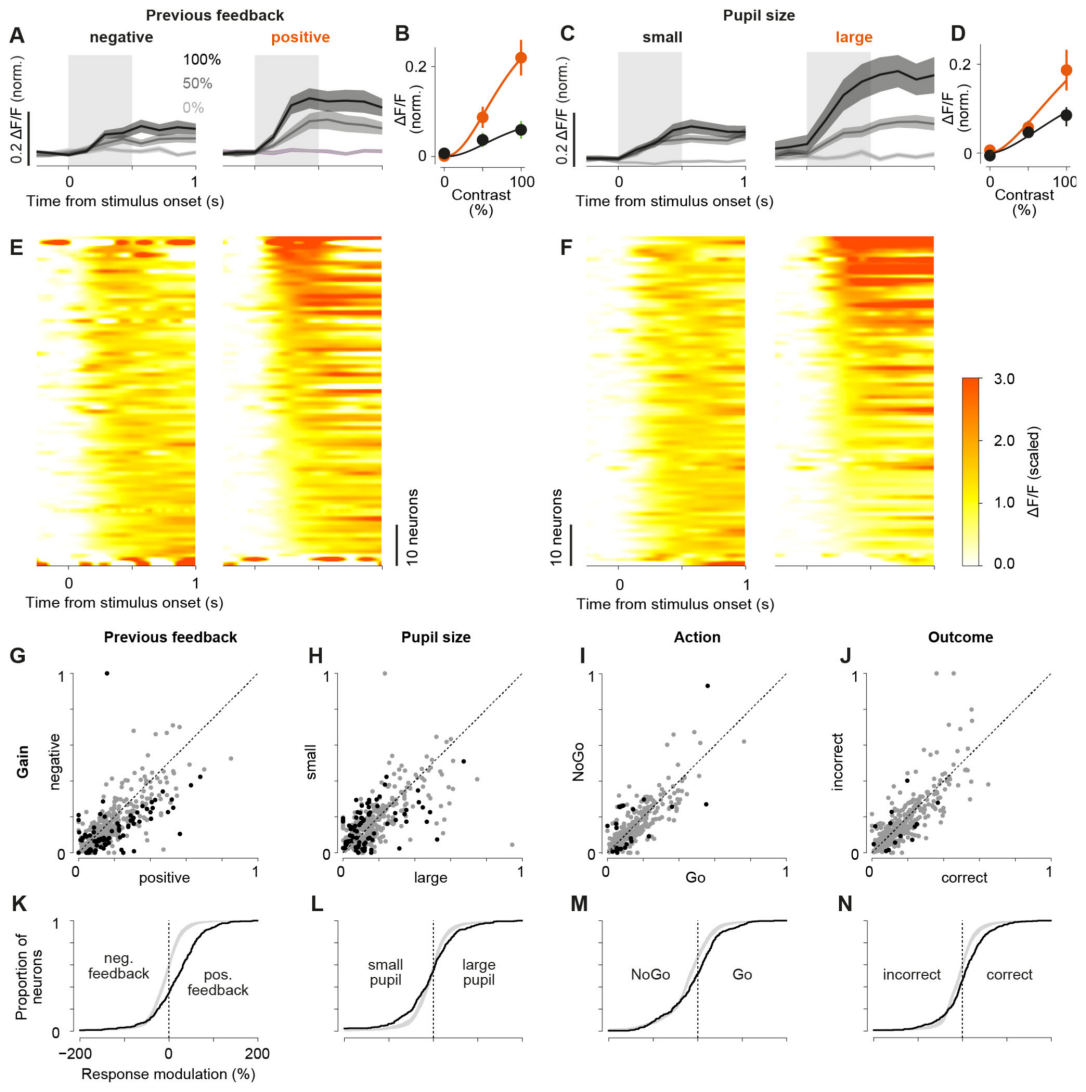
Visual responses in the superficial superior colliculus are modulated by previous feedback and pupil size. **A**. Calcium traces of sSC neuron in response to visual stimuli of different contrasts after animal received negative (*left)* or positive (*right)* feedback. Shaded region: 0.0–0.5 s after stimulus onset, window used to determine response amplitudes (in **B,D**). Horizontal line: at 0 ΔF/F. Scale bar: 0.2 ΔF/F (recorded traces were scaled to range from 0 to 1). **B**. Visual responses (same neuron as in **A**) after negative (black) or positive (orange) feedback, fitted with a hyperbolic ratio function. Responses were baseline subtracted (mean of 0–0.5 s before stimulus onset). **C,D**. Similar as **A,B** for a different neuron (same scalebar). Responses were split according to pupil size. **E**. Mean calcium traces in response to 100% contrast stimuli after negative (*left*) or positive (*right*) feedback. Only neurons with significant modulation by previous feedback are shown (80 neurons). Traces were scaled so that mean responses to negative feedback equal 1. **F**. Same as in **E** but for modulation by pupil size (79 neurons). **G-J**. Gain of visually responsive neurons (407) for previous feedback (**G**), pupil size (**H**), action (**I**), and outcome (**J**). Black dots: significantly different gains (p < 0.05, permutation test). Neurons with significantly increased/decreased responses: 63/17 following reward (**G**), 32/47 during large pupil (**H**), 17/17 during Go trials (**I**), 8/5 before correct choices (**J**). **K-N**. Cumulative distributions of response modulation (black line). Grey shade: 2.5th to 97.5th interval of null distribution.

Visual responses of sSC neurons were most strongly affected by previous feedback and changes in pupil size (Figure 2). We quantified tuning to visual contrast by fitting a hyperbolic ratio function to each neuron’s responses to contralateral stimuli (Figure 2A-D). If this fit explained the data better than a flat line, the neuron was considered *visually responsive*. For these 407 (of 497) neurons, we determined the modulation of visual responses by four task variables: feedback received on the previous trial (“previous feedback”, positive or negative), pupil size (small or large), upcoming action (Go or NoGo), and upcoming outcome (correct or incorrect). To explain the impact of any of these variables on the contrast tuning curve, we varied the gain of the contrast tuning curve while keeping all other variables constant. We modelled the gain by a weighted sum of all four variables and, for each neuron, determined which of the four variables contributed significantly to the modulation of its gain. The model including the significant variables (*best model*) was used in all following analyses if not stated otherwise. Examples of neurons modulated by previous feedback and pupil size are shown in Figure 2A-D. In total, 80 of 407 neurons (20%) were significantly modulated by previous feedback (Figure 2E) while 79 of 407 neurons (19%) were modulated by pupil size (Figure 2F). To assess the impact of each task variable, we compared the fitted contrast gain between both values of each task variable, e.g., the fitted gain after negative versus positive feedback (Figure 2G). Gains for one variable were determined using each neuron’s best model with all other variables set to zero. If the best model of a neuron did not include the variable (grey dots in Figure 2G-J), we refitted the model now including that variable. We quantified the impact of each variable by its *response modulation (RM)*: the difference between both gains (e.g., negative and positive feedback), normalized by their mean (Figure 2K-N). A response modulation of 0% means that both gains were the same, while the maximum of 200% means that one of the gains is zero and the other is >0. Of all visually responsive neurons, 41% (169 of 407) were modulated by at least one task variable. Previous feedback affected 20% of neurons (80 of 407, 14% ± 1%, 6 mice; Figure 2G,K). Reward, i.e., positive feedback, mostly increased visual responses by an average of 78% (out of significant RMs: 79% positive RMs: 0.56 mean, equivalent to a 1.8x modulation; 21% negative RMs: -0.60 mean, equivalent to a 0.5x modulation). Pupil size, on the other hand, affected 19% of neurons (79 of 407; 17% ± 1%, 6 mice; Figure 2H,L) causing increases (out of significant RMs: 41% positive RMs: 0.39 mean, equivalent to a 1.5x modulation) and decreases (out of significant RMs: 59% negative RMs: -0.78, equivalent to a 0.4x modulation) of visual responses. These effects are likely to reflect modulation by pupil-linked arousal as has been observed before (Schröder et al., 2020). However, we cannot exclude additional modulation by changes in retinal illumination due to changes in pupil size. Finally, upcoming action (Figure 2I,M) and outcome (Figure 2J,N) modulated less than 6% of the neurons (action: 24 of 407, outcome: 13 of 407).

The rate of modulation by previous feedback and pupil size was not significantly different for excitatory and inhibitory neurons. To differentiate between excitatory and inhibitory neurons, we used a mouse line resulting from crossing the Gad2-IRES-Cre and the Ai9 Cre reporter lines to express the red fluorescence TdTomato in GAD2-expressing cells. While very similar proportions of excitatory and inhibitory neurons were affected by previous feedback (excitatory: 15% ± 14%, inhibitory: 15% ± 13%; 16 sessions in four mice; Χ^2^ = 0.03, p = 0.88), the effect of pupil size on excitatory and inhibitory populations was more variable but not significantly different (excitatory: 19% ± 15%, inhibitory: 9% ± 16%; 16 sessions in four mice; Χ^2^ = 0.28; p = 0.28).

**Figure 3:**
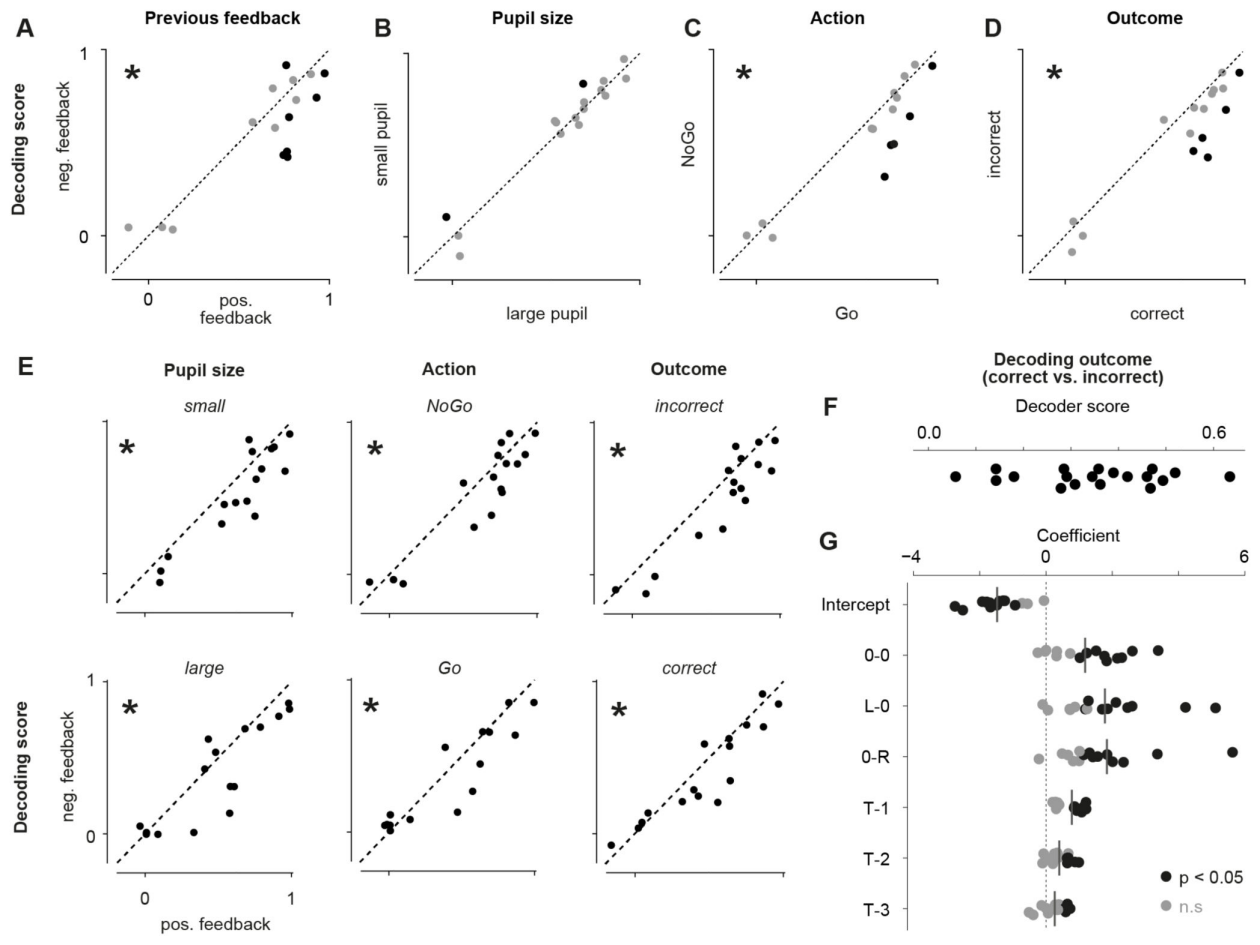
Decoding from visual responses improves after reward. **A**. Decoding scores of logistic regression models to detect presence of visual stimulus contralateral to the recorded SC neurons, tested on trials after positive versus negative feedback. Significant difference in predictive power per dataset (black dots) and across datasets (star) determined with permutation test (p < 0.05, 16 sessions in four mice). **B-D**. Same as in **A**, but trials were split by different task variables. **E**. Decoding scores for neural responses after positive versus negative feedback, only considering trials with fixed pupil size (*left*), fixed action (*middle*), or fixed outcome (*right*). Significant score differences across sessions marked by star (p < 0.05, permutation test, 16 sessions in four mice). **F**. Decoding scores of logistic regression models trained to predict trial outcome (correct or incorrect) based on visual stimulus in the current trial (0-0, L-0, or 0-R) and outcome of the previous three trials (T-1, T-2, T-3). All trained models performed better than chance (p < 0.05, permutation test, 16 sessions in four mice). **G**. Coefficients of logistic regression models to predict trial outcome. Black: coefficients are significantly different from 0 (p < 0.05, Wald test). Grey lines show medians of coefficients across sessions. In 10 of 16 sessions, reward in the previous trial increased the likelihood of correct outcome (see “T-1”).

Decoding the presence of a visual stimulus from populations of sSC neurons was improved in trials following reward compared to trials following negative feedback (Figure 3A-E). We trained a logistic regression decoder on each neuronal population recorded in 16 sessions (four mice) to detect contralateral stimuli based on peri-stimulus activity. The selected sessions included trials where no contralateral stimulus was presented, which was not the case for the six sessions disregarded in this analysis. The decoder was trained on a subset of trials and then tested on the held-out trials grouped by either previous feedback, pupil size, upcoming outcome, or upcoming action. Decoding performance was better after reward but was not influenced by pupil size (Figure 3A,B). Decoding performance was also improved before correct choices and Go actions (Figure 3C,D). We ensured that these differences in decoding performance were not caused by an imbalance of stimulus contrasts between conditions. The distributions of stimulus contrasts were similar across values of each task variable. To account for the small differences, we balanced the data when training and testing the decoder. Secondly, we wanted to ensure that correlations between task variables did not confound this analysis. Indeed, animals were more likely to make a correct choice after a rewarded compared to an unrewarded trial (P(correct | positive feedback) = 0.69 ± 0.01, P(correct | negative feedback) = 0.59 ± 0.03, p < 0.01, t-test). Go actions, however, were as likely after rewarded and unrewarded trials (P(Go | positive feedback) = 0.75 ± 0.04, P(Go | negative feedback) = 0.74 ± 0.04, p = 0.78, t-test). To control for the lack of independence between task variables, we repeated the decoding analysis for each task variable while fixing another task variable to one value. We found that the improved decoding performance from visual responses after rewarded trials could not be explained by correlations with pupil size, action, or outcome (Figure 3E). In contrast, outcome and action had no effect on decoding performance when only trials following negative feedback were considered.

Task performance was higher in trials following reward (Figure 3F,G). To test whether previous reward had behavioral consequences, we used a logistic regression decoder to predict the outcome of a trial (correct or incorrect) based on stimulus contrast and outcome of the previous three trials. The results show that trial outcome could be predicted above chance (Figure 3F) and that reward on the previous trial increased the likelihood of a correct choice in addition to the effect of stimulus contrast (Figure 3G). The improved decoding performance of stimulus presence in the population of sSC neurons following rewarded trials could be a reason for the improved task performance. However, changes in neurons downstream of sSC are likely to affect task performance as well.

**Figure 4:**
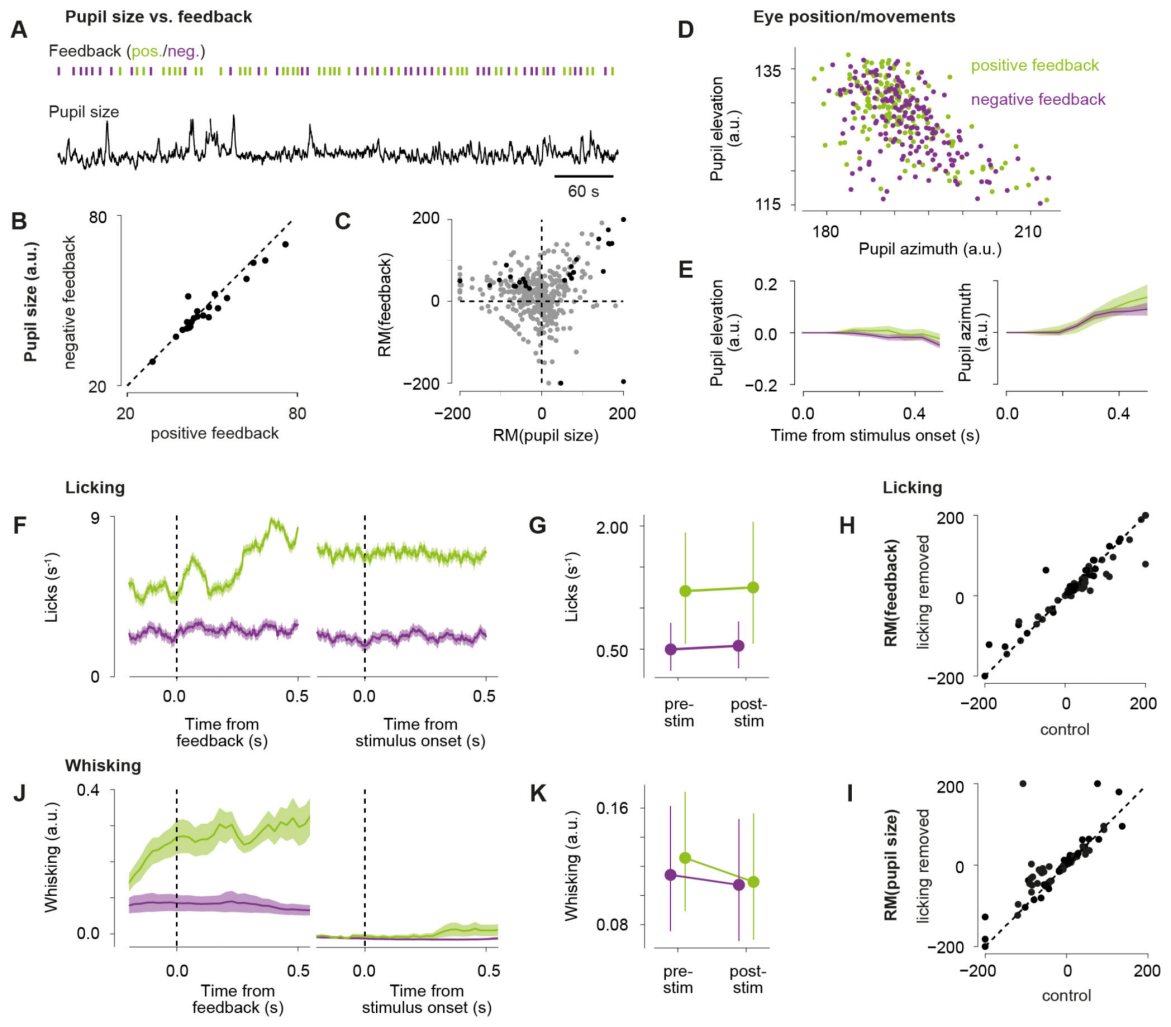
Modulation by previous feedback cannot be explained by changes in pupil size, eye position or licking. **A**. Example trace of pupil size and trial outcomes. **B**. Pupil size (mean across trials) following positive versus negative feedback (t(21) = 1.523, p = 0.143, paired t-test). **C**. Response modulation (RM) by pupil size versus previous feedback (same data as shown in Figure 2K,L). Black dots: pupil size and previous feedback significantly contributed to gain modulation. Tests: all data: Pearson’s r = 0.23, p < 0.05, 407 neurons; black dots only: Pearson’s r = 0.20, p = 0.30, 27 neurons. **D**. Eye positions at time of stimulus onset after animal received positive (green) or negative (purple) feedback during one task session (241 and 213 trials). **E**. Eye movements in vertical (*left*) and horizontal (*right*) direction at time of stimulus onset after animal has received positive or negative feedback. **F**. Lick rate locked to feedback onset (*left*) and stimulus onset (*right*) following positive or negative feedback (241 and 213 trials, 1 example session). **G**. Lick rate (18 sessions) before (-0.5–0 s) and after (0–0.5 s) visual stimulus onset following positive or negative feedback. Mice licked more following rewarded than non-rewarded trials (F(1,64) = 6.25, p < 0.05), but lick rate was not significantly different between pre- and post-stimulus periods (F(1,64) = 0.002, p = 0.95). Test: two-way ANOVA (factors: time (pre-/post-stimulus) x feedback). **H,I**. Response modulation (RM) by previous feedback (**H**) and pupil size (**I**) for all trials (“control”) versus for no-lick trials (no licks in response window). RMs were not significantly different (feedback: z = 0.59, p = 0.56, 80 neurons; pupil size: z = 1.76, p = 0.08, 79 neurons; LMM). Only neurons significantly modulated by previous feedback or pupil size were considered. **J,K**. Same as in **F,G** but for whisking. Whisking was not significantly different between pre- and post-stimulus periods (F(1,64) = 0.27, p = 0.60, 17 sessions in four mice). Test: two-way ANOVA (factors: time (pre-/post-stimulus) x feedback).

While both previous feedback and pupil size modulated visual responses, they did so independently of each other (Figure 4A-C). To test whether effects by pupil size and reward had a common underlying cause, we compared pupil size during stimulus presentation after positive and negative feedback and found no difference (Figure 4A,B). This shows that previous feedback had no significant impact on pupil size measured several seconds after the feedback was provided. In support of this conclusion, significant modulations by pupil size and previous feedback were independent of each other (Figure 4C, black dots).

Modulation by reward and pupil size could not be explained by licking, eye position, or whisking (Figure 4D-K). We found that reward delivery induced a higher lick rate compared to negative feedback, and that the lick rate after reward stayed high beyond the beginning of the next trial (Figure 4F). However, lick rate stayed constant before and after the onset of the visual stimulus (Figure 4G). As visual responses were baseline subtracted, i.e., activity before stimulus onset was subtracted from activity after stimulus onset, it is unlikely that licking caused larger visual responses following positive feedback. Similarly, lick rates were higher during large pupil trials but did not change around the time of stimulus onset. To control for any effects related to licking, we repeated analyses while removing trials in which the animal licked during the tested time window. This did not affect the observed modulation by reward or pupil size (Figure 4H,I). Other than lick rate, eye position and whisking were not affected by reward at the time the following visual stimulus was presented.

Variations in eye position at the time of stimulus onset directly affects RF-stimulus overlap and thus the visual drive experienced by the neuron. While this initial eye position varied across trials, previous reward had no consistent effect on initial eye position (Figure 4D) and the distributions of eye positions after positive versus negative feedback shared the same center of mass (p > 0.05, permutation test). In addition, pupil movements during stimulus presentation showed no difference following positive versus negative feedback (azimuth: z = 0.014, p = 0.093, elevation: z = -1.504, p = 0.133, LMM); Figure 4E). In an additional group of four mice (same as used below for electrophysiology experiments), we recorded a video of the animal’s face whileperforming the visual decision task and quantified whisking based on the movement energy of the whisker pad. While we observed anticipatory whisking activity prior to reward, which continued after reward delivery, whisking activity during stimulus presentation was the same whether or not the previous trial was rewarded (Figure 4J,K). Whisking activity, eye position, and eye movements during stimulus presentation were also not affected by pupil size (eye position: p > 0.05, permutation test; eye movements: azimuth: z = 1.681, p = 0.093, elevation: z = -1.493, p = 0.135, LMM; whisking for small versus large pupil trials: F(1,64) = 5.28, p < 0.05, two-way ANOVA for pre-/post-stimulus time x pupil size, 17 sessions in four mice).

**Figure 5:**
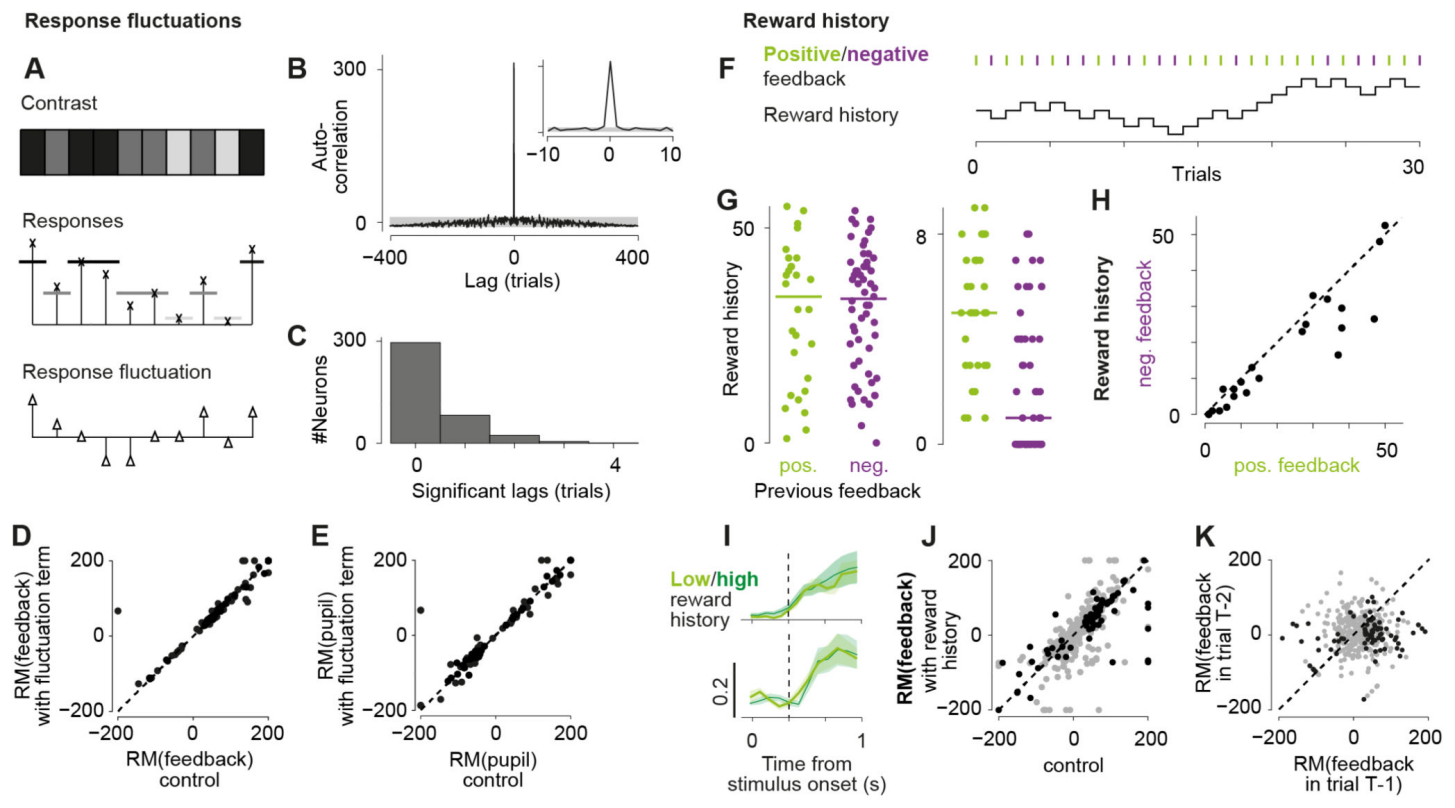
Modulation by previous feedback is independent of slow response fluctuations and reward history. **A**. Quantification of visual response fluctuation (see *Response fluctuation analysis* in *Materials and Methods)*. **B**. Auto-correlogram of fluctuation trace (mean across visually responsive neurons in one session). Grey shade: 2.5th to 97.5th percentile interval of null distribution. Inset: same data on smaller x-axis. **C**. Histogram of largest absolute lags with significant correlation strengths. **D,E**. Response modulation (RM) by previous feedback (**D**) and pupil size (**E**) as determined previously (“control”) and when accounting for response fluctuations. RMs were not significantly different (feedback: z = -0.238, p = 0.81, 99 neurons; pupil size: z = -0.193, p = 0.85, 89 neurons; LMM). Only neurons significantly modulated by previous feedback or pupil size were considered. **F**. Quantification of cumulative reward history. **G**. Cumulative reward history for trials with previously positive and negative feedback for two example sessions (*left* and *right*). Median reward histories (horizontal lines) are different in the right example (p < 0.0001, t-test). **H**. Median reward history across trials with previously positive or negative feedback. Trials with previously positive reward had slightly more positive reward histories (z = 4.803, p = 0.001, LMM). **I**. Calcium traces of two example neurons aligned to stimulus onset for trials following reward. Trials with reward histories larger than the median (dark green) or smaller than the median (light green) were pooled. **J**. Response modulation (RM) by feedback as determined previously (“control”) and when accounting for reward history. RMs were not significantly different (z = -0.293, p = 0.77, 407 neurons, LMM). Black dots: neurons with significant feedback modulation. **K**. Response modulation (RM) by feedback in the previous trial (T-1) and by feedback experienced two trials earlier (T-2). Black dots: neurons with significant feedback (in T-1) modulation (80 neurons). RMs for T-1 were significantly different from 0 (t = 3.21, p < 0.01, t-test), whereas RMs for T-2 were not (t = 0.60, p = 0.54, t-test).

Reward modulation of visual responses was independent of slow response fluctuations (Figure 5A-E). Task engagement and motivation can affect both task performance and neural responses across the duration of an experimental session (Jacobs et al., 2020; Ortiz et al., 2020), which may explain the observed reward modulation. To quantify slow fluctuations of visual responses, we measured trial-to-trial variations from mean responses to each stimulus contrast (Figure 5A) and calculated the auto-correlogram of this stimulus-independent activity (Figure 5B). Hardly any neuron showed consistent response fluctuations exceeding two consecutive trials indicating that visual responses did not exhibit slow fluctuations (Figure 5C). As a further test, we included stimulus-independent activity from the previous trial as additional predictor variable when fitting the gain of the contrast tuning curve. This did not diminish the impact of reward in the previous trial or of pupil size on the visual response (Figure 5D,E). Task engagement and alertness may also be reflected in the action of wheel turning. However, the effect of previous feedback did not change when we restricted our analysis to visual responses that followed NoGo trials, i.e., the mouse did not move the wheel (z = -0.038, p = 0.97, LMM). This shows that wheel turns during the previous trials could not explain the modulation by previous reward.

Lastly, modulation by previous reward could not be explained by an association between the visual stimulus and the reward learned during the session; instead it was specific to visual responses immediately following the reward (Figure 5F-K). Our previous analyses focused on the modulation of the visual responses by the feedback that immediately *preceded* the visual stimulus. In contrast, most previous studies on reward modulations reported that sensory responses change when the animal learns that a sensory stimulus predicts a reward, i.e., reward *succeeds* the stimulus (also see Discussion). Although the animals in this study have been trained extensively before neural responses were recorded, we investigated whether reward history *within* the recording session could explain our findings. In other words, did the repeated experience of receiving reward after seeing the contralateral stimulus (in correct trials) increase the visual response to the contralateral stimulus? To quantify reward history, we considered all trials showing the contralateral stimulus (any contrast above 0%) and then subtracted the number of incorrect from the number of correct trials that occurred before the current trial (Figure 5F). Across all sessions, reward history was significantly different between trials following positive versus negative feedback (Figure 5G,H). However, the maximum difference between median reward histories was only 8.5 trials and, in six of 16 sessions, reward history was not significantly different between trials following positive versus negative feedback (p < 0.05, t-test). This is a strong indication that reward history could not explain the modulation of visual responses immediately following reward. We then tested how reward history influenced visual responses. As shown for two example neurons modulated by reward, visual responses after positive reward were the same following high and low reward histories (Figure 5I). When we included reward history as a fifth variable to model the gain of the contrast tuning curves, response modulations by previous feedback did not change significantly (Figure 5J). Finally, to test the time scale of reward effects, we modelled the gain of the contrast tuning curve by the weighted sum of the four variables pupil size, previous feedback, action, and outcome, but replaced the variable for feedback in the previous trial by feedback two trials before the visual stimulus. We found that the number of neurons modulated by feedback two trials before was largely reduced compared to feedback in the directly preceding trial (Figure 5K). This finding highlights the temporal specificity of reward modulation.

**Figure 6:**
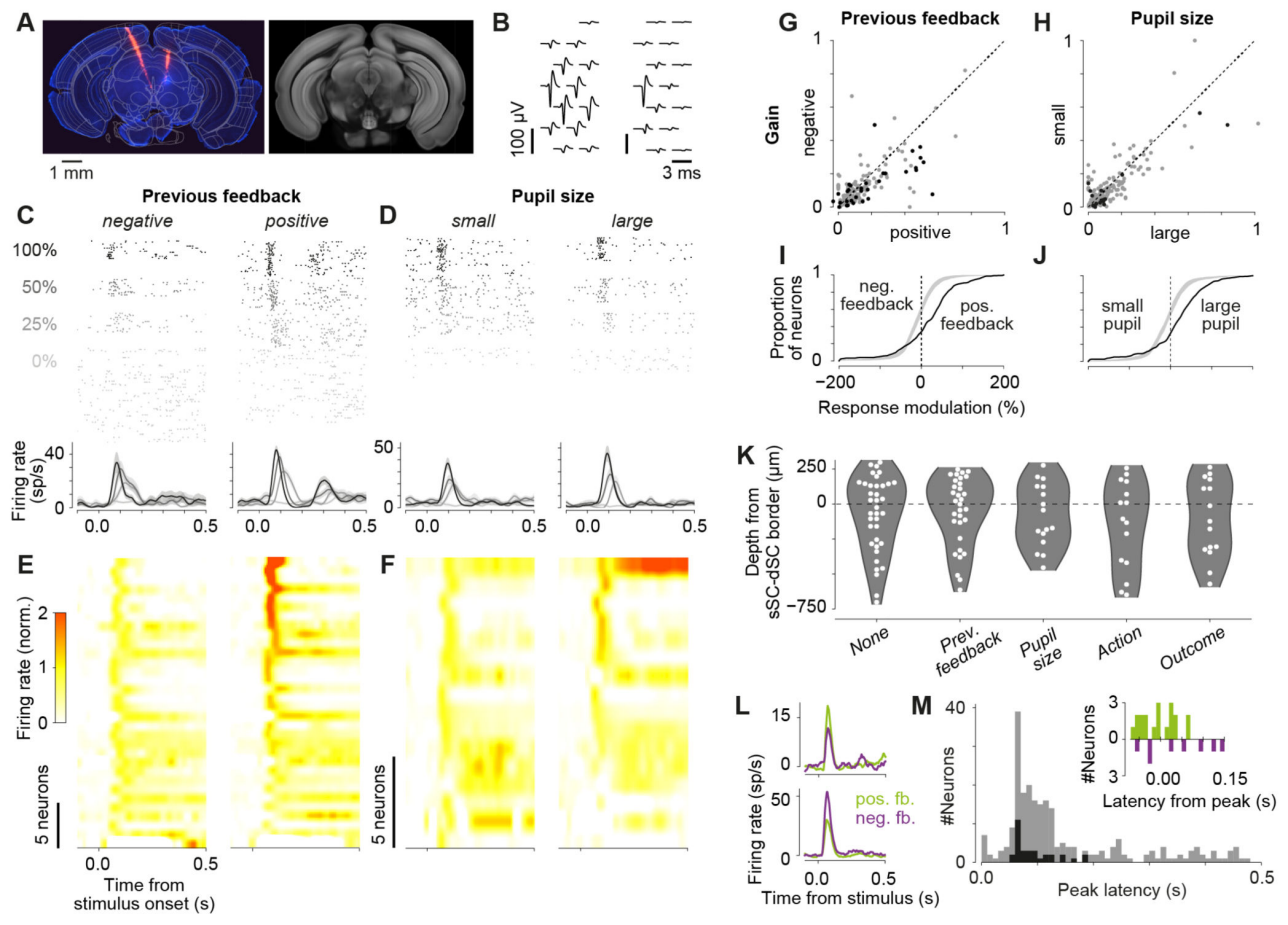
Modulation by previous feedback extends to deep SC and affects early phase of visual responses. **A**. Coronal brain slice (DAPI staining) with tracks of electrophysiological probes (red) and overlay of brain area borders (*left)*. Border between sSC and dSC was estimated at 310 μm below SC surface. Section of Allen CCF mouse atlas aligned to brain slice (*right*). **B**. Spike waveforms recorded on nearby channels of the Neuropixels probe for two sSC neurons. **C,D**. Spike times (*top*) and firing rate (*bottom*) aligned to stimulus onset (from same neurons as in **B**) after animal received negative (*left*) or positive (*right*) feedback. **E**. Firing rates in response to 100% contrast stimuli after negative (*left*) or positive (*right*) feedback. Only neurons with significant modulation by previous feedback are shown (32 neurons). **F**. Same as in **E** but for modulation by pupil size (16 neurons). **G,H**. Gain of visually responsive neurons (158, 15 sessions in four mice) for previous feedback (**G**) and pupil size (**H**). Black dots: significantly different gains (p < 0.05, permutation test). Neurons with significantly increased/decreased responses: 24/8 following reward (**G**), 12/4 during large pupil (**H**). **I,J**. Cumulative distributions of response modulation (black line). Grey shade: 2.5th to 97.5th interval of null distribution. **K**. Depth of neurons within SC modulated by different task variables. Number of significantly modulated sSC/dSC neurons: 16/16 by previous feedback, 6/10 by pupil size, 7/8 by action, and 6/9 by outcome. Dashed line: border between superficial and deep superior colliculus. **L**. Firing rates of two neurons aligned to stimulus onset after positive or negative feedback. **M**. Latency of peak visual response for neurons significantly modulated by previous feedback (black) and other neurons (gray). Inset: Earliest significant difference in firing rates after positive versus negative feedback, relative to response peak (p < 0.05, t-test with Bonferroni-Holm correction). Only neurons with significantly larger responses after positive (green) or after negative (purple) feedback are considered.

Using electrophysiological recordings, we confirmed that visual responses in the sSC are modulated by previous reward and pupil size (Figure 6A-K). We trained four additional mice in a very similar task as described before: two Gabor patches with contrasts between 0% to 100% appeared in the left and right hemifield and the mouse had to move the higher contrast patch towards the center; only when both patches had 0% contrast, the wheel had to be kept still. Using Neuropixels 1.0 probes, we recorded single neurons across the depth of SC (15 sessions in four mice). We used Kilosort 2.5 to automatically detect and sort spikes and manually curated the resulting clusters based on spike amplitude, variance of spike amplitude across time, spike auto-correlogram, and other features (see *Materials and Methods* for details, examples in Figure 6B). We determined the surface of the SC along the probe based on the shape of the visually evoked local field potential. To estimate the location of the border between superficial and deep SC as well as the depth of the SC, we reconstructed the location of the probe aligning histological brain slices to the Allen Mouse Brain Common Coordinate Framework (Figure 6A). In total, we were able to record 1,037 neurons in the SC (416 in sSC, 621 in dSC). Based on the neurons’ responses across stimulus contrast, we found that 158 neurons were visually responsive. Among these neurons, 32 (20%) were significantly modulated by previous feedback and 16 (10%) by pupil size (Figure 6C-F). Similar to the results above, previous reward mostly increased visual responses by an average of 123% (out of significant RMs: 75% positive RMs: 0.76 mean, equivalent to a 2.2x modulation; 25% negative RMs: -0.83 mean, equivalent to a 0.4x modulation; Figure 6G,I). In contrast to the earlier results, pupil size mostly increased visual responses (out of significant RMs: 75% positive RMs: 0.85 mean, equivalent to a 2.5x modulation; 25% negative RMs: -0.93 mean, equivalent to a 0.4x modulation; Figure 6H,J). This may be explained by a difference between superficial and deep layers, however, the number of recorded neurons is too small for a definitive conclusion (response increase in pupil modulated neurons: sSC: 4 of 6 neurons, 67%; dSC: 8 of 10 neurons, 80%). A comparison between superficial and deep layers shows that the proportion of visually responsive neurons was very similar between sSC and dSC (sSC: 74 of 416 neurons, 18%; dSC: 84 of 621 neurons, 14%), and so was the modulation by previous reward (sSC: 16 of 74 neurons, 22%; dSC: 16 of 84 neurons, 19%) and pupil size (sSC: 6 of 74 neurons, 8%; dSC: 10 of 84 neurons, 12%; Figure 6K).

Modulation by previous reward coincided with the peak visual response approximately 80 ms after stimulus onset (Figure 6L,M). The high temporal resolution afforded by electrophysiological recordings revealed the precise timescale of visual response modulation by reward. In two example neurons, previous reward took effect at the time of the onset of the visual response with the largest effect around the peak of the response, no matter whether reward increased or decreased the response (Figure 6L). This was representative of the rest of the neurons affected by reward. The mean latency of their peak response occurred 78 ± 8 ms after stimulus onset, and the earliest significant difference between responses after reward versus negative feedback occurred from 70 ms before to 150 ms after the peak (Figure 6M).

**Figure 7:**
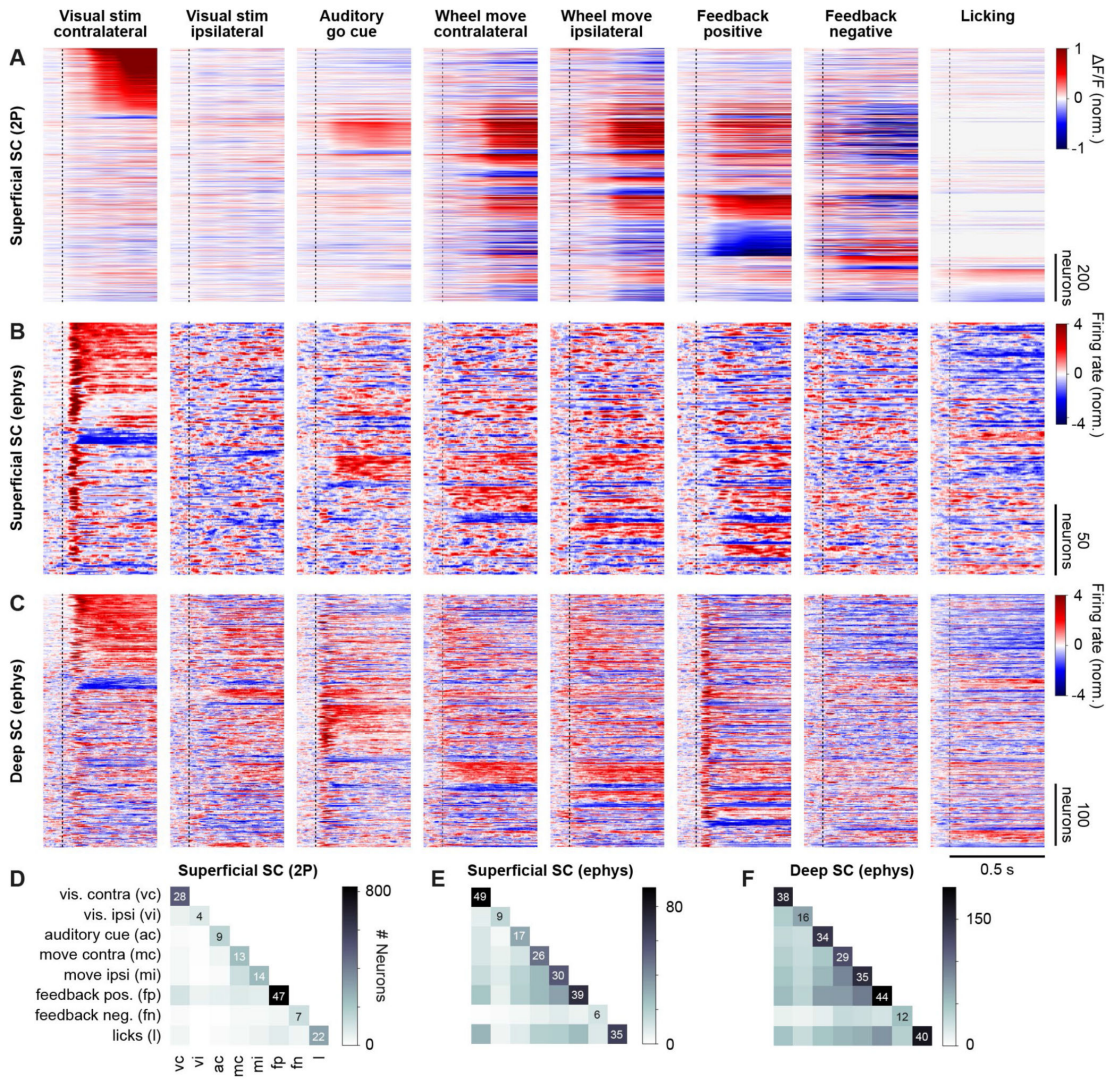
SC neurons respond to various task events. **A.** Mean calcium traces (traces were z-scored across recording) of 1,751 task responsive neurons locked to onsets (dashed lines) of the visual stimulus (at 100% contrast), auditory go cue, wheel move, feedback, and licking. Order of neurons is the same across all plots. Neurons were first grouped by the first event they responded to (order of tested events as shown in plot), and then sorted by response amplitude. Responses to licking (*last column*) shown for 269 neurons (18 sessions, five mice); lick times not recorded for the rest of the sessions (indicated by light gray). **B,C**. Similar to **A**, but for mean firing rates of 184 task responsive sSC neurons (**B**) and 422 task responsive dSC neurons (**C**) recorded using electrophysiology. In **A-C**, traces were first z-scored across each recording. **D**. Number of sSC neurons (recorded with two-photon imaging) with significant responses to a specific task event (*diagonal*, numbers are percentage of task responsive neurons) or pairs of task events (*below diagonal*). Note that lick data were recorded for a subset of 2,902 neurons. **E,F**. Similar to **D**, but for sSC (**E**) and dSC (**F**) neurons recorded using electrophysiology.

In addition to modulation by reward and pupil size, a large proportion of neurons in the sSC responded to non-visual task events (Figure 7). We tested whether any of the neurons recorded using two-photon imaging and electrophysiology had significant responses (p < 0.05, t-test or ANOVA) to the following events: onset of the contra- and ipsilateral task stimuli, the auditory go cue, ipsi- and contralateral wheel turns in no-stimulus trials, positive and negative feedback in no-stimulus trials, and licks outside the reward delivery period (Figure 7A-C). Around 50% of all recorded neurons responded to at least one of these events, termed *task responsive neurons* (sSC with two-photon imaging: 1,751 of 3,919, 45%; sSC with electrophysiology: 184 of 316, 58%; dSC: 422 of 721, 58%). The non-visual events eliciting responses in the largest number of task responsive neurons were reward, licking, wheel turns, and the auditory go cue (Figure 7D-F). Ipsilateral visual stimuli and negative feedback elicited responses in the fewest number of neurons. Around 60% of the neurons responding to contralateral stimuli also responded to other events (sSC-2P: 63%, sSC-ephys: 55%, dSC: 68%), showing that many neurons in the SC, and particularly in the sSC, not only respond to visual input. It was therefore essential to control for the impact of these task events when studying the modulation of visual responses, as we have done above. Interestingly, relatively few neurons whose visual response was modulated by previous reward responded to reward itself (sSC-2P: 11%, sSC-ephys: 31%, dSC: 18%), or to negative feedback (sSC-2P: 6%, sSC-ephys: 6%, dSC: 0%).

## Discussion

We found that visual responses of about 20% of neurons in mouse superficial SC were modulated directly after the animal received a reward for making a correct choice in a visual decision task. This reward modulation improved the ability of a decoder to detect a visual stimulus based on the neural population activity, which indicates that the reward modulation may help the animal to detect a visual stimulus and thus perform better in a visual detection task. Accordingly, behavioral performance was better following correct, rewarded trials. Visual responses of just under 20% of sSC neurons were also modulated by pupil size, a common read-out of arousal. Modulation by pupil size was, however, independent of reward modulation and did not improve decoding of stimulus presence. Neither modulation by reward nor pupil size could be explained by other behaviors (licking, whisking, or eye movements), slow fluctuations in neural activity, or accumulated reward history. We observed these results using two-photon imaging of calcium concentration and confirmed our findings using electrophysiology in a different set of animals.

Modulation of visual responses by previous reward has not been described before. Investigations on the effects of reward on sensory and specifically visual responses have focused on stimulus-reward associations. In stimulus-reward association paradigms, the animal perceives a stimulus and is then given a reward, either depending on a behavioral response from the animal (goal-directed task) or independent of it (conditioning). After many repetitions, usually across several days, the animal learns that the stimulus predicts the reward, which is reflected in the animal’s behavior, e.g., the animal starts licking as soon as it perceives the stimulus but before a water reward is delivered. In case of visual stimuli, it has been shown that neurons in primary visual cortex increase their responses to the rewarded stimulus compared to their response when the stimulus was not predictive of reward (Goltstein et al., 2013, 2018; Poort et al., 2015; Jurjut et al., 2017; Henschke et al., 2020). Similar effects on visual responses due to stimulus-reward associations have been described in deep layers of the SC (Ikeda and Hikosaka, 2003; Griggs et al., 2018; Woolrych et al., 2021) but not in superficial layers. Similar to V1, visual responses of some deep SC neurons increased if the visual stimulus predicted a reward, while other deep SC neurons increased responses to control movements towards a rewarded target (Felsen and Mainen, 2012). While the animals in the current study underwent goal-directed task learning, the reward modulation we report is of a different nature. Here, visual responses increased due to the immediately preceding reward, not due to a learned stimulus-reward association and independent of how often the stimulus was rewarded in the current session (reward history). Reward modulation was also independent of the reason the reward was delivered, i.e., whether the animal correctly responded to a contra- or ipsilateral stimulus or no stimulus at all. The described reward modulation occurred in the superficial, visual as well as the deeper layers of SC. It was different from previous findings of neural responses to reward itself (Steinmetz et al., 2019; Szadai et al., 2022). Although some neurons in SC responded at the time of reward, this response usually subsided before the presentation of the next visual stimulus. Any remaining differences in baseline were subtracted when determining visual responses.

We observed that visual responses in the SC were modulated following a rewarding experience (drop of water), but was it the reward itself inducing this modulation or a change of attentional state? Rewarding experiences powerfully influence visual attention (Chelazzi et al., 2013; Anderson, 2016; Failing and Theeuwes, 2018), which makes it very difficult to disentangle effects of reward versus visual attention on neural activity and behavioral performance (Maunsell, 2004). Furthermore, the SC implements a priority map, which integrates visual information with task-relevant information (Veale et al., 2017), and plays a key role in controlling visual attention (Krauzlis et al., 2013; Basso and May, 2017; Basso et al., 2021). A recent study performed a change detection task in mice and found that neurons in the intermediate layers of SC increased their visual response if the location of the visual stimulus was cued (Wang et al., 2022). The same processes that achieve such attentional effects may also underlie the reward modulation we observed here. To advance our understanding of the impact exerted by attention, it is, however, necessary to carefully define what we mean by attention. One type refers to spatial attention, which is described as a mental spotlight enhancing the processing of the illuminated item (Posner et al., 1980) or which biases competition between several objects in the visual input towards the one currently relevant for behavior (Desimone and Duncan, 1995). Our observations are not consistent with such spatial selectivity because no spatial location was cued in our task paradigm. Instead, our observations reflect a global enhancement of visual input independent of location, as the reward modulation was independent of the position of the previously rewarded stimulus and even the presence of a stimulus in the rewarded trial. It remains to be seen whether the reward modulation is specific to stimulus features, like feature-specific attention. If not, it may be more suitable to treat reward modulation as a different behavioral state that is not reflected by changes in pupil size.

Receiving a water drop as a reward is also concomitant with the quenching of thirst, and across many trials can decrease motivation and task engagement (Flavell et al., 2022). Could this explain the observed reward modulation? In mice performing a Go-NoGo task, responses to auditory Go cues across many brain regions generally diminished when the mouse became sated towards the end of a task session (Allen et al., 2019). These slow dynamics occurring across tens of minutes cannot explain the reward modulation observed here, which was independent of slow response fluctuations and was determined by reward delivered only seconds before the visual response.

We showed that reward increased responses to subsequent visual stimuli, which led to better stimulus detectability (using a decoder). But what is the advantage of increased stimulus detectability after receiving a reward? We hypothesize that a reward could prompt the animal to expect more reward in the same context, so that improved perception of the environment would help the animal to recognize this context again. This prompting is not necessary when the animal is performing our task paradigm. In a natural environment, in contrast, opportunities for reward may be rare and sensory input following this reward may indicate further reward that can be found in the same or similar environment (more apples on the same tree or more apples on other apple trees). Increased stimulus detectability after reward could even help to learn an association between these stimuli with reward (Dommett et al., 2005; Eldar et al., 2013; Takakuwa et al., 2017). Interestingly, some SC neurons project to the ventral tegmental area (VTA) or substantia nigra (Comoli et al., 2003; Fields et al., 2007; Solié et al., 2022), which are part of the reward circuit of the brain thought to form stimulus-reward associations. It is hypothesized that these SC neurons projecting to dopamine neurons may provide visual information to reward circuits, which learn reward predictions based on this visual information (Redgrave et al., 2010).

Further open questions relate to the necessary behavioral context for the observed reward modulation and the underlying mechanisms. In this study, the animal was engaged in a visual decision task, in which it had to perform the correct action following a visual stimulus to receive a reward. Is such a paradigm necessary to invoke modulation of visual responses by previous reward? According to our current working hypothesis, reward increases responses to the following visual stimulus to improve the perception and subsequent recognition of the visual environment, in which the reward was collected. We therefore expect that task engagement or active reward-association learning is not necessary for the observed reward modulation. Instead, we expect that even randomly delivered reward will increase responses to subsequent visual stimuli. But how does information about reward reach the superior colliculus? We think that the most promising sources are neuromodulators, which are emitted after reward delivery acting on local circuits across the timespan of seconds. This could explain the effect of reward on the early visual response (close to peak response) compared to effects on later phases (several hundreds of milliseconds after stimulus onset), which would be more consistent with feedback mechanisms triggered by the visual stimulus. The superficial layers of the SC receive projections from serotonergic neurons in the dorsal raphe nucleus and dopaminergic neurons in the zona incerta, and express receptors for both neuromodulators (Beitz et al., 1986; Shukla et al., 2014; Bolton et al., 2015; Benavidez et al., 2021). The dorsal raphe nucleus and the zona incerta exhibit changes in activity with reward (Cohen et al., 2015; Sizemore et al., 2020; Wang et al., 2020b) and could transmit reward related information to the sSC.

## Figures and Tables

**Table 1:** Descriptions and statistics of datasets. Abbreviations: 2P – two-photon imaging, ephys – electrophysiology with Neuropixels probes, UFC-1St – two-alternative unforced choice task with at most one visual stimulus per trial, AFC-2St – two-alternative forced choice task with two visual stimuli per trial, UCF-2St – two-alternative unforced choice task with two stimuli per trial, #recorded – number of recorded neurons, #visual – number of visually responsive neurons, #reward – number of neurons modulated by previous feedback, #pupil – number of neurons modulated by pupil size.

Mode	Task	Animal	Date	#visual	#reward	#pupil
2P	UFC-1St	SS041	2015-08-11	14	1	3
		SS047	2015-11-10	29	4	5
			2015-11-11	1	0	0
			2015-11-17	0	0	0
			2015-11-18	9	3	2
		SS048	2015-08-20	15	1	0
			2015-08-27	16	3	1
			2015-09-03	31	1	2
			2015-09-16	39	5	3
			2015-09-26	37	4	5
		SS052	2015-11-24	20	2	4
			2015-12-01	14	2	2
			2015-12-02	46	14	10
			2015-12-03	55	17	18
			2015-12-04	17	5	1
			2015-12-15	24	3	6
	AFC-2St	SS057	2016-06-29	4	0	0
			2016-07-01	8	0	1
			2016-07-06	17	2	0
		SS059	2016-06-29	22	2	1
			2016-07-07	24	3	3
			2016-07-09	55	8	12
Ephys	UFC-2St	SS087	2017-12-12	42	6	4
			2017-12-13	17	0	0
		SS088	2018-01-30	4	0	1
			2018-01-31	15	1	0
			2018-02-01	10	1	0
		SS089	2018-02-06	32	4	2
			2018-02-07	20	2	1
			2018-02-08	11	1	0
			2018-02-09	14	2	1
			2018-02-10	10	0	0
			2018-02-11	14	1	0
		SS093	2018-05-24	26	4	2
			2018-05-25	24	8	3
			2018-05-26	5	0	1
			2018-05-27	6	2	1

## Data Availability

The pre-processed data generated in this study are available at https://doi.org/10.25377/sussex.c.6208999; code used to analyze pre-processed data is available at https://github.com/liadJB/Baruchin-et-al-2023. The raw data are available on reasonable request.

## Significance Statement

To learn which actions lead to food, water, or safety, it is necessary to integrate the receiving of reward with sensory stimuli related to the reward. Cortical stages of sensory processing have been shown to represent stimulus-reward associations. Here we show however that reward influences neurons at a much earlier stage of sensory processing—the superior colliculus—receiving direct input from the retina. Visual responses were increased shortly after the animal received the water reward, which led to an improved stimulus signal in the population of these visual neurons. Reward modulation of early visual responses may thus improve perception of visual environments predictive of reward.
